# QTL discovery for resistance to black spot and cercospora leaf spot, and defoliation in two interconnected F1 bi-parental tetraploid garden rose populations

**DOI:** 10.3389/fpls.2023.1209445

**Published:** 2023-07-27

**Authors:** Jeekin Lau, Haramrit Gill, Cristiane H. Taniguti, Ellen L. Young, Patricia E. Klein, David H. Byrne, Oscar Riera-Lizarazu

**Affiliations:** Department of Horticultural Sciences, Texas A&M University, College Station, TX, United States

**Keywords:** black spot, cercospora leaf spot, defoliation, quantitative trait loci, *Rosa*

## Abstract

Garden roses are an economically important horticultural crop worldwide, and two major fungal pathogens, black spot (*Diplocarpon rosae* F.A. Wolf) and cercospora leaf spot of rose (*Rosisphaerella rosicola* Pass.), affect both the health and ornamental value of the plant. Most studies on black spot disease resistance have focused on diploid germplasm, and little work has been performed on cercospora leaf spot resistance. With the use of newly developed software tools for autopolyploid genetics, two interconnected tetraploid garden rose F_1_ populations (phenotyped over the course of 3 years) were used for quantitative trait locus (QTL) analysis of black spot and cercospora leaf spot resistance as well as plant defoliation. QTLs for black spot resistance were mapped to linkage groups (LGs) 1–6. QTLs for cercospora resistance and susceptibility were found in LGs 1, 4, and 5 and for defoliation in LGs 1, 3, and 5. The major locus on LG 5 for black spot resistance coincides with the previously discovered *Rdr4* locus inherited from *Rosa* L. ‘Radbrite’ (Brite Eyes™), the common parent used in these mapping populations. This work is the first report of any QTL for cercospora resistance/susceptibility in tetraploid rose germplasm and the first report of defoliation QTL in roses. A major QTL for cercospora susceptibility coincides with the black spot resistance QTL on LG 5 (*Rdr4*). A major cercospora resistance QTL was found on LG 1. These populations provide a genetic resource that will further the knowledge base of rose genetics as more traits are studied. Studying more traits from these populations will allow for the stacking of various QTLs for desirable traits.

## 1 Introduction

**Figure 1:**
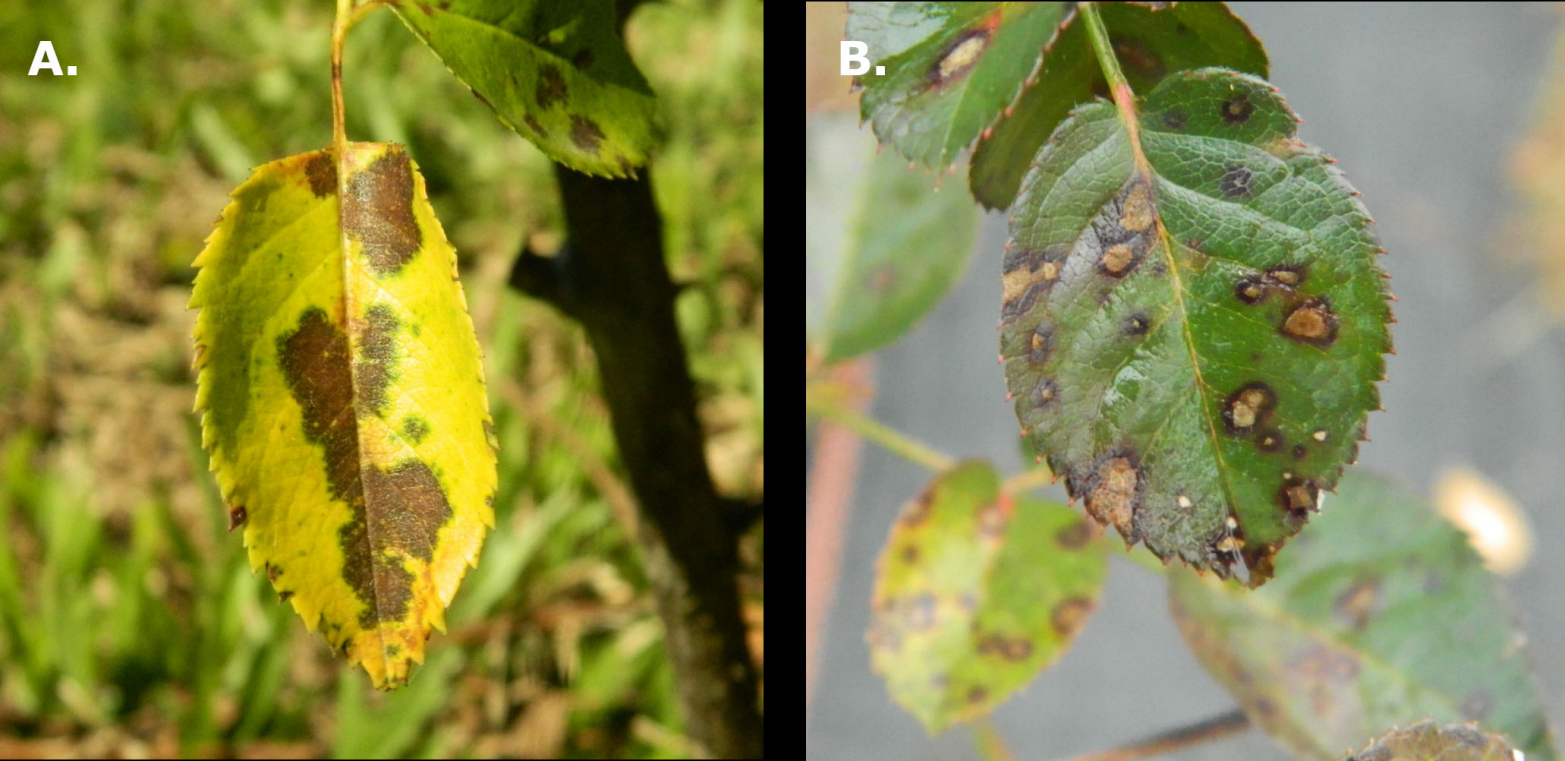
**(A)** Black spot of rose on a leaf resulting in chlorosis on a rose leaflet. **(B)** Cercospora leaf spot on a rose leaflet.

In the United States, garden roses had $168 million in sales in 2019 (USDA-NASS, 2019). Host plant disease resistance in garden roses (*Rosa* spp.) is among the most important traits to both breeders and consumers, as the general public favors the reduction of chemical pesticide use. Thus, breeders are focused on the development of resistant commercial cultivars, as these cultivars require fewer fungicide applications throughout the growing season (Debener and Byrne, 2014; Byrne et al., 2019; Waliczek et al., 2018; Chavez et al., 2020). Black spot (*Diplocarpon rosae* F.A. Wolf) and cercospora leaf spot of rose (*Rosisphaerella rosicola* Pass. previously known as *Cercospora rosicola* Pass.) are two important fungal pathogens, both of which can cause defoliation. Black spot is ubiquitous in warm humid regions. Black spot infections cause black lesions on leaves with feathery borders that spread, causing chlorosis of the leaves around the lesion border (**Figure 1A**) (Horst and Cloyd, 2007). Heavy infestations of black spot can completely defoliate a susceptible plant, resulting in both weak and visually unappealing plants. Cercospora leaf spot infects plants from the older growth and progresses to the newer growth, causing circular lesions with necrotic tan or gray centers (**Figure 1B**) (Horst and Cloyd, 2007; Mangandi and Peres, 2018). These two diseases will be referred to simply as black spot and cercospora. Defoliation can be caused by these two diseases discussed above as well as abiotic heat-induced stress.

Garden roses have complex genetics, as they are the result of interspecific crosses among roughly 10 *Rosa* spp. and can be diploid (2*n* = 2*x* = 14), triploid, and tetraploid (Zlesak, 2007). Roses are generally outcrossing and highly heterozygous. Much of the genetic work performed in roses has been focused on resistance to black spot, resulting in the discovery of four vertical resistance genes/loci (*Rdr1*, *Rdr2*, *Rdr3*, and *Rdr4*) and quantitative trait loci (QTL) for horizontal resistance (Xue and Davidson, 1998; Von Malek et al., 2000; Hattendorf et al., 2004; Whitaker et al., 2007; Whitaker et al., 2010; Dong et al., 2017; Zurn et al., 2018; Yan et al., 2019; Zurn et al., 2020; Rawandoozi et al., 2022). In addition, a recent meta-QTL analysis revealed black spot resistance QTLs on linkage groups (LGs) 3 and 5 across multiple diploid mapping populations (Lopez Arias et al., 2020). The majority of the black spot resistance genes and QTL were discovered using diploid germplasm except for *Rdr3* and *Rdr4*, which were mapped in two tetraploid populations (Zurn et al., 2018; Zurn et al., 2020). The existence of multiple black spot races, with differential virulence, in combination with fluctuating weather conditions, which can alter the severity and progression of the disease, are obstacles in studying this disease. In contrast to black spot resistance, very little work has been performed on the genetics of cercospora resistance and defoliation. Previous cercospora work showed that it was difficult to replicate cercospora infection in a greenhouse (Kang, 2020). Work on cercospora resistance in garden roses has only been reported recently at the diploid level (Rawandoozi et al., 2023). Without the availability of selective fungicides, it is difficult to study both pathogens separately, as both black spot and cercospora are present in our fields. Additionally, black spot infections can defoliate plants, making it hard to score plants for cercospora. Defoliation is affected by both biotic and abiotic stresses (Sakamoto et al., 2008), thus possibly presenting difficulties in studying its inheritance.

QTL studies are used to identify genomic regions affecting traits, and their accuracy relies on the usage of high-quality linkage maps and phenotypic data. Currently, there are seven high-density single-nucleotide polymorphism (SNP)-based linkage maps in tetraploid roses. Five of these maps were genotyped using the WagRhSNP 68k array (Bourke et al., 2017; Zurn et al., 2018; Zurn et al., 2020; Lau et al., 2022), and two other maps were genotyped using specific-locus amplified fragment sequencing (SLAF-seq) markers (Cheng et al., 2021; Yu et al., 2021). New and actively developed linkage mapping software such as the R-packages “polymapR” (Bourke et al., 2018) and “MAPpoly” (Mollinari and Garcia, 2019) allows for efficient linkage mapping using thousands of SNP markers. QTL mapping software packages like “polyqtlR” (Bourke et al., 2021) and “QTLpoly” (Pereira et al., 2020) take the outputs of the linkage mapping software for subsequent QTL mapping. The continued development of these new software packages has allowed us to better study the genetics of polyploid crops.

To extend our knowledge on black spot resistance, address the gap in research on cercospora resistance, and investigate the genetic factors affecting defoliation in tetraploid garden roses, we used two tetraploid garden rose F_1_ populations. This study used multiple QTL mapping software to 1) identify regions of interest affecting black spot resistance, cercospora resistance, and defoliation; 2) use QTL results to identify individuals and haplotypes useful for breeding with these populations; and 3) incorporate findings in this study with previous research to select individuals carrying multiple disease resistance loci for use in breeding.

## 2 Materials and methods

### 2.1 Population development

Two F_1_ bi-parental tetraploid garden rose families, *Rosa* L. ‘ORAfantanov’ (Stormy Weather™) × *Rosa* L. ‘Radbrite’ (Brite Eyes™) (SWxBE) n = 200 and *Rosa* L. ‘Radbrite’ (Brite Eyes™) × *Rosa* L. ‘BAIgirl’ (Easy Elegance® My Girl) (BExMG) n = 157, were developed in 2016 as a joint effort between Texas A&M University Rose Breeding and Genetics Program and Weeks Roses. These populations were previously described and used for mapping rose rosette disease resistance (Lau et al., 2022). The genotypes were clonally propagated via rooted cuttings. The rooted cuttings were then planted in a randomized complete block design with one plant per rep and two reps per genotype at the Texas A&M University Horticulture Teaching Research and Extension Center in Somerville, TX (30.52, −96.42) in 2018. Black landscape fabric was used for weed suppression, and overhead irrigation was installed to encourage black spot and cercospora development. No fungicides were sprayed. The soil type is Belk clay, and plants were fertilized and irrigated as needed. Plants were pruned yearly during a dormant stage to a uniform size of around 0.5 m^3^.

### 2.2 Phenotyping

Plants were phenotyped monthly for black spot, cercospora, and defoliation incidence from June to November 2019, from May to August 2020, and from May to September 2021. Data were recorded using Field Book (Rife and Poland, 2014). Black spot, cercospora, and defoliation were visually rated on a scale of 0–9 in which 0 represents no disease in a canopy or a lack of defoliation. A rating of 1 would be representative of a plant that had 1%–10% of the leaves with disease lesions or 1%–10% of the leaves missing. A rating of 2 would represent 11%–20% of leaves with disease lesions or leaves missing, etc.

### 2.3 Variance component estimates

Linear mixed models were used to estimate the best linear unbiased estimates (BLUEs) used for QTL mapping and phenotypic variance due to genotype, environment (each set of observations), and reps nested within the environment, using the Restricted Estimated Maximum Likelihood (REML) method in R package ASReml-R version 4.1 (Butler et al., 2009). Mixed models using REML methods are better at estimating model effects when there are random missing data, which is common in field research (Holland et al., 2003). Mixed models also allow us to more accurately estimate the performance of each genotype given environmental differences. Variance component estimates for black spot, cercospora, and defoliation were estimated using a completely random model:


$$y_{ijk}=\;\mu +G_{i}+E_{j}+R_{k\left(j\right)}+GE_{ij}+\varepsilon_{ijk},$$

where $y_{ijk}$ is the phenotypic value of genotype *i* at environment *j* in block *k*, μ is the overall mean, $G_{i}$ is the effect of genotype *i*, $E_{j}$ is the effect of the environment *j* (monthly observation set), $R_{k\left(j\right)}$ is the effect of a block nested within an environment, $GE_{ij}$ is the genotype by environment effect, and $\varepsilon_{ijk}\;$ is the residual defined as the interaction between the plot and correlation of monthly observations (autocorrelation of measuring the same plant in the field multiple times). For estimating BLUEs used in QTL scans, the effect of genotype was considered fixed, while the rest of the model was considered random. The same mixed model was used to calculate BLUEs for each year and BLUEs for all the observations together.

### 2.4 Genotyping, linkage mapping, and QTL mapping

Unexpanded young leaf tissues were collected, flash-frozen in liquid nitrogen, and stored at −80°C until DNA extraction. DNA was extracted using a cetyl trimethylammonium bromide (CTAB) protocol (Yan et al., 2018). Genotyping was performed using the WagRhSNP 68K Axiom SNP array, and linkage mapping steps were performed previously (Lau et al., 2022). Phased linkage maps constructed in R package polymapR (Bourke et al., 2018) were imported into R package MAPpoly (Mollinari and Garcia, 2019) to estimate genotypic probabilities for each progeny used for QTL mapping in R package QTLpoly (Pereira et al., 2020). Phenotypic values used in the QTL scans were BLUEs calculated from the mixed models mentioned above. QTL scans were conducted using the random-effect multiple interval mapping (REMIM) methods. The REMIM method first builds a null model (no QTL model), followed by rounds of forward search of QTL and backward elimination of QTL. The genome-wide significance levels (α = 0.2 and α = 0.05) used for the forward search of QTL and backward eliminations were determined by using a score-based resampling method to establish a genome-wide significance by simulating QTL at every position in the linkage map (Zou et al., 2004; Pereira et al., 2020). The simulation was run 1,000 times prior to QTL mapping to obtain the p-values to be used for forward search and backward elimination of QTL in the REMIM method. After rounds of adding and eliminating QTL, the model of the location of the QTL was refined, and confidence intervals were calculated for each QTL. Estimates of the QTL’s effect on the phenotypic mean in QTLpoly were calculated using the “qtl_effects” function.

MAPpoly estimates the parental haplotypes inherited by each progeny at every map position. Thus, in the QTL mapping process, if we identify a QTL position and the parental homolog that donates the favorable allele to the trait, we are able to identify individuals with the QTL carrying this specific homolog segment. This allows the selection of individuals with multiple favorable QTL alleles and enables the efficient stacking of disease resistance QTL. Monthly, yearly, and overall BLUEs resulted in 57 QTLs that were visualized with the newly developed R Shiny application VIEWpoly (Taniguti et al., 2022). This application facilitates the interactive investigation of our QTL results and creates useful graphics and tables that allow the user to efficiently mine the QTL scanning data to quickly identify QTL of interest and subsequently select individuals that carry the QTL for breeding. We used VIEWpoly to quickly aggregate our results into tables and produce visual aids for plotting our QTL and their effects. Candidate gene searches were conducted for regions under the QTL using the Genome Database for Rosaceae’s “Search Genes and Transcripts” tool (Jung et al., 2019). The genotypic data, phenotypic data, and linkage maps are archived on Genome Database for Rosaceae under publication datasets under the accession number tfGDR1068 (https://www.rosaceae.org/publication_datasets).

### 2.5 Joint family analysis

In addition to the bi-parental populations used for the QTL studies, another approach leveraging the interconnectedness of these two populations allowed for a joint QTL analysis. A Julia software package PolyOrigin (Version 0.5.10) (Zheng et al., 2021) was used to reconstruct the parental haplotypes in all the progeny together. Thus, the theoretical resolution of the common parental haplotypes should be better, as there are more recombination breaks observed in the two interconnected populations compared to only one by itself. The joint family QTL analysis was performed using the R package diaQTL (version 1.08) (Amadeu et al., 2021). The QTL peaks were then reported as corroborating evidence for the presence of those QTLs.

## 3 Results

### 3.1 Variance components and correlations

**Figure 2:**
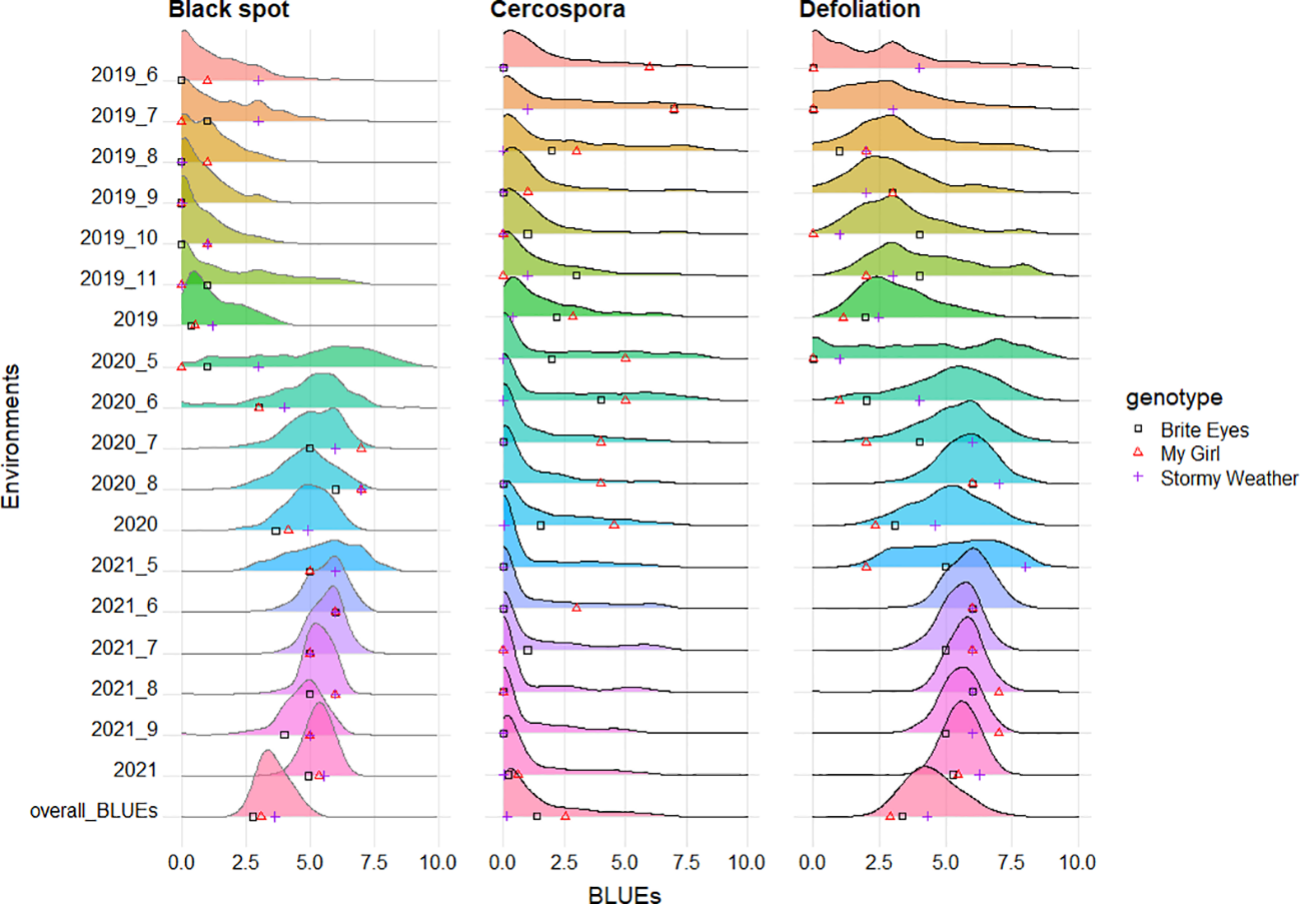
Phenotypic distributions of black spot, cercospora, and defoliation, taken in two bi-parental tetraploid mapping populations phenotyped over 3 years in Somerville, TX. The distributions present phenotypic best linear unbiased estimates (BLUEs) of each genotype by environment (month), year, or overall data. The parental genotypic BLUEs are plotted on the X-axis of each graph. A number after a given year (2019, 2020, and 2021) indicates the month when data were collected (6 = June, 7 = July, 8 = August, 9 = September, 10 = October, and 11 = November). A year without an additional number presents BLUEs across months for a given year. Overall_BLUEs presents BLUEs from analysis across months and years.

Black spot, cercospora, and defoliation were phenotyped over 15 months (environments) over the course of 3 years. We considered each month a different environment due to varying weather conditions creating different disease pressure on the plants. Black spot ratings had a low level (4%) of phenotypic variation attributed to genotypic effects, whereas cercospora (49%) and defoliation (18%) ratings had higher amounts of variation attributed to genetic factors (**Table 1**). A large portion of phenotypic variance for black spot and defoliation was from the environment (63 and 30%, respectively), while cercospora had only 6% of its variance attributed to the environment. Black spot resistance and defoliation were variable across environments, while the expression of cercospora resistance was more uniform over all environments (**Figure 2**). Genotype by environment variance ranged from 12% to 23% for all traits. The autocorrelation of monthly data from the same plot was minimal for all traits (**Table 1**).

**Table 1:** Sources of variation and heritability estimates from two tetraploid garden rose mapping populations, Brite Eyes × My Girl and Stormy Weather × Brite Eyes phenotyped for black spot, cercospora leaf spot, and defoliation in Somerville, TX, in 2019, 2020, and 2021.

	Black spot ^a^	% ^b^	Cercospora	%	Defoliation	%
Genotype	0.26 (0.03)***	4.27	2.47 (0.20)***	48.91	0.88 (0.08)***	17.66
Environment	3.96 (1.51)***	62.81	0.31 (0.12)***	6.19	1.51 (0.59)***	30.23
Rep (Environment)	0.05 (0.02)***	0.82	0.01 (0.00)***	0.29	0.09 (0.03)***	1.71
Genotype × Environment	0.77 (0.03)***	12.26	0.59 (0.03)***	11.70	1.13 (0.04)***	22.58
Autocorrelation	0.00 (0.00)	0.00	0.12 (0.01)***	2.34	0.05 (0.00)***	1.16
Residual	1.25 (0.03)***	19.89	1.55 (0.03)***	30.57	1.33 (0.03)***	26.67

a Variance components followed by standard errors in parentheses.

b Percent of phenotypic variance explained.

*** Variance components are significant at p ≤ 0.001 using likelihood ratio test.

**Figure 3:**
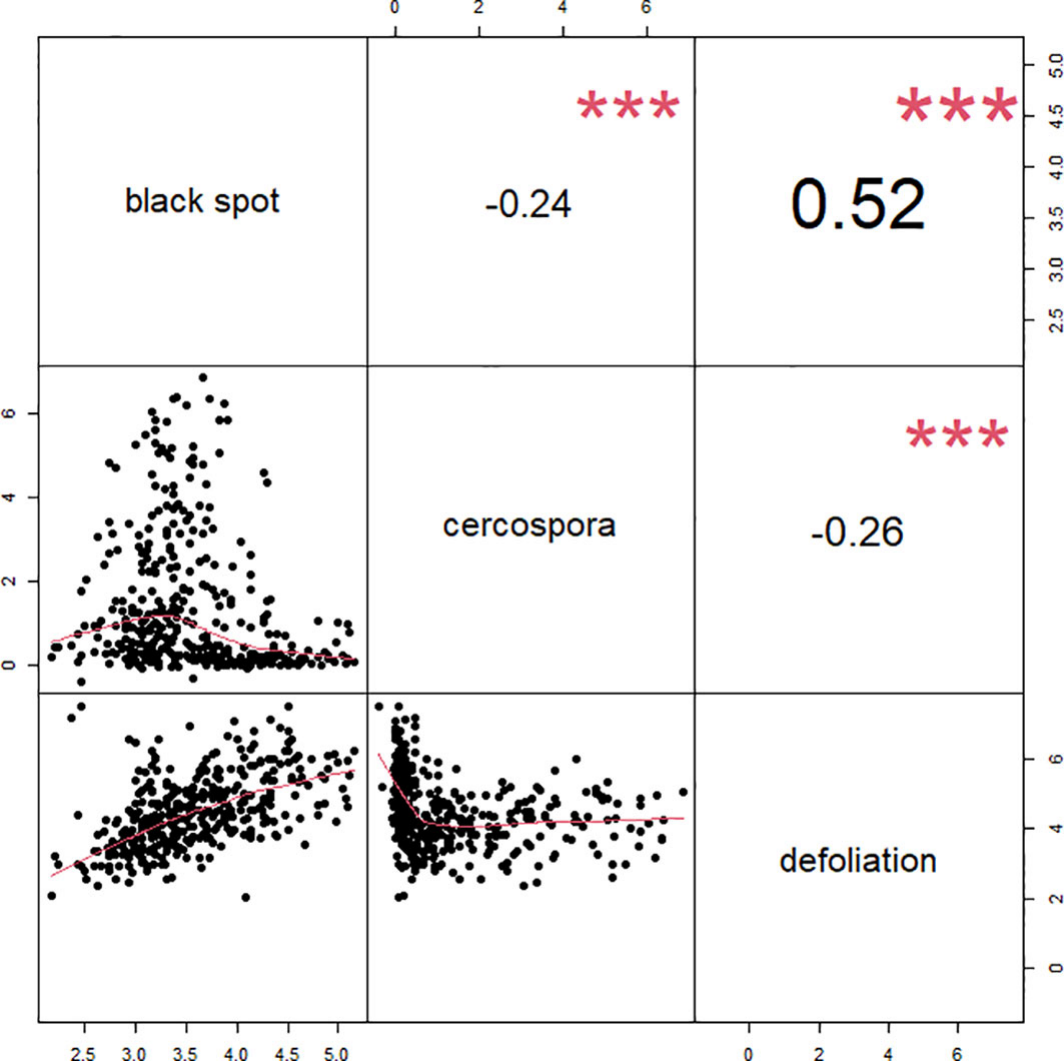
Correlations between phenotypic best linear unbiased estimates (BLUEs) for each genotype across three traits black spot, cercospora, and defoliation, for 3 years of data and across phenotyped in two tetraploid garden rose populations in Somerville, TX.

A moderate positive correlation was observed between black spot and defoliation (0.52), and low negative correlations were observed between cercospora and black spot (−0.24) and between cercospora and defoliation (−0.26) (**Figure 3**). High correlations were observed in the phenotypic scores from year to year for cercospora (0.84–0.90) and moderate correlations for defoliation (0.60–0.72), while there was a low year-to-year correlation for black spot (0.15–0.21) (**Supplementary Figure 1**). The distribution of phenotypic scores of each trait as the experiment progressed over time shows that there was a change in distribution, through time, of black spot incidence and defoliation (**Figure 2**). As time progressed, there was a loss of resistant phenotypes for black spot, but the distribution of phenotypic scores remained relatively constant for cercospora.

### 3.2 QTL mapping

**Figure 4:**
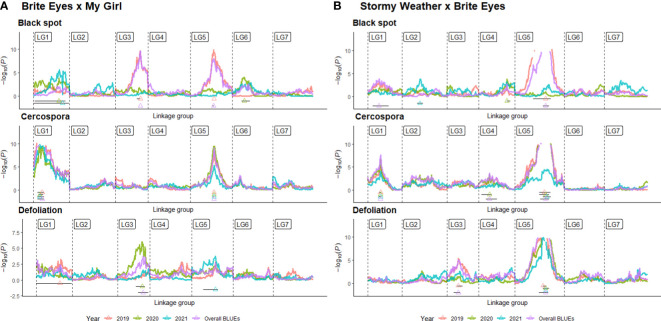
Quantitative trait locus (QTL) profiles of black spot and cercospora resistance/susceptibility and defoliation phenotyped in Somerville, TX, over 3 years in two bi-parental tetraploid garden rose mapping populations **(A)** Brite Eyes x My Girl, and **(B)** Stormy Weather x Brite Eyes. QTL profiles generated from QTL scans of yearly best linear unbiased estimates (BLUEs) and overall BLUEs in QTLpoly and figures generated using VIEWpoly.

**Figure 5:**
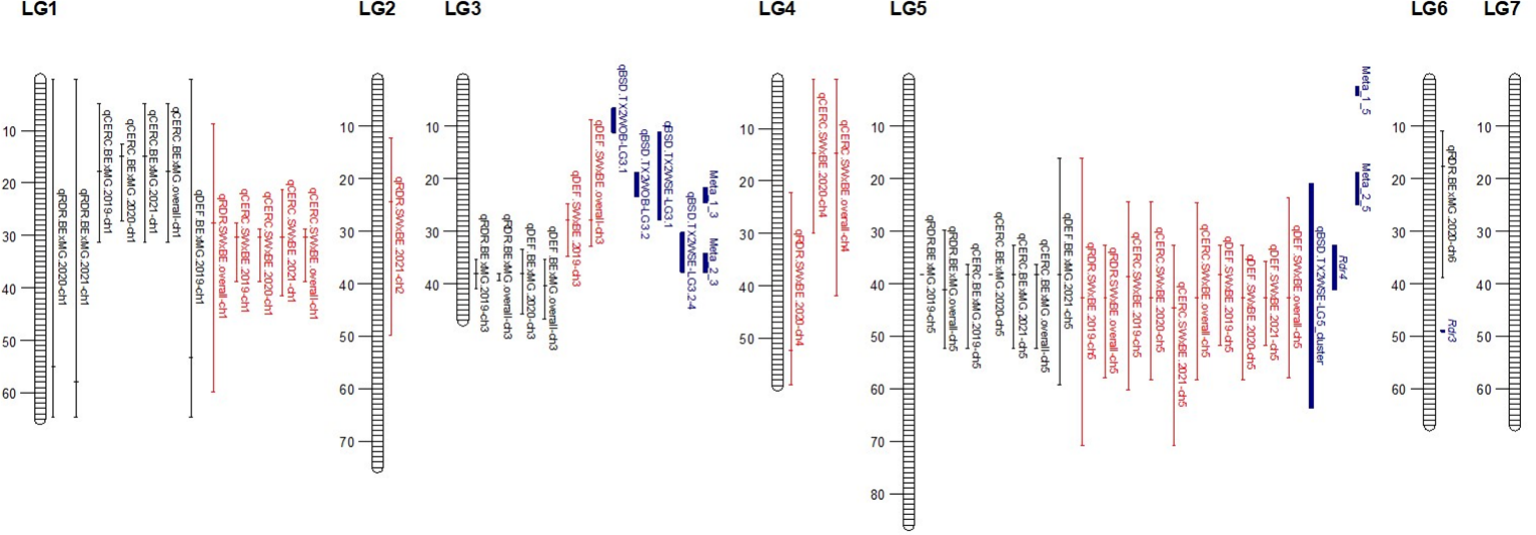
Quantitative trait locus (QTL) for black spot and cercospora resistance/susceptibility and defoliation detected in two interconnected tetraploid garden rose mapping populations phenotyped in Somerville, TX, over 3 years. Positions are in Mbp. QTL peaks denoted as a tick mark on the bars and are color coded for the two mapping populations Brite Eyes × My Girl (black) Stormy Weather × Brite Eyes (red) and previously reported loci and meta-QTLs (blue).

**Figure 6:**
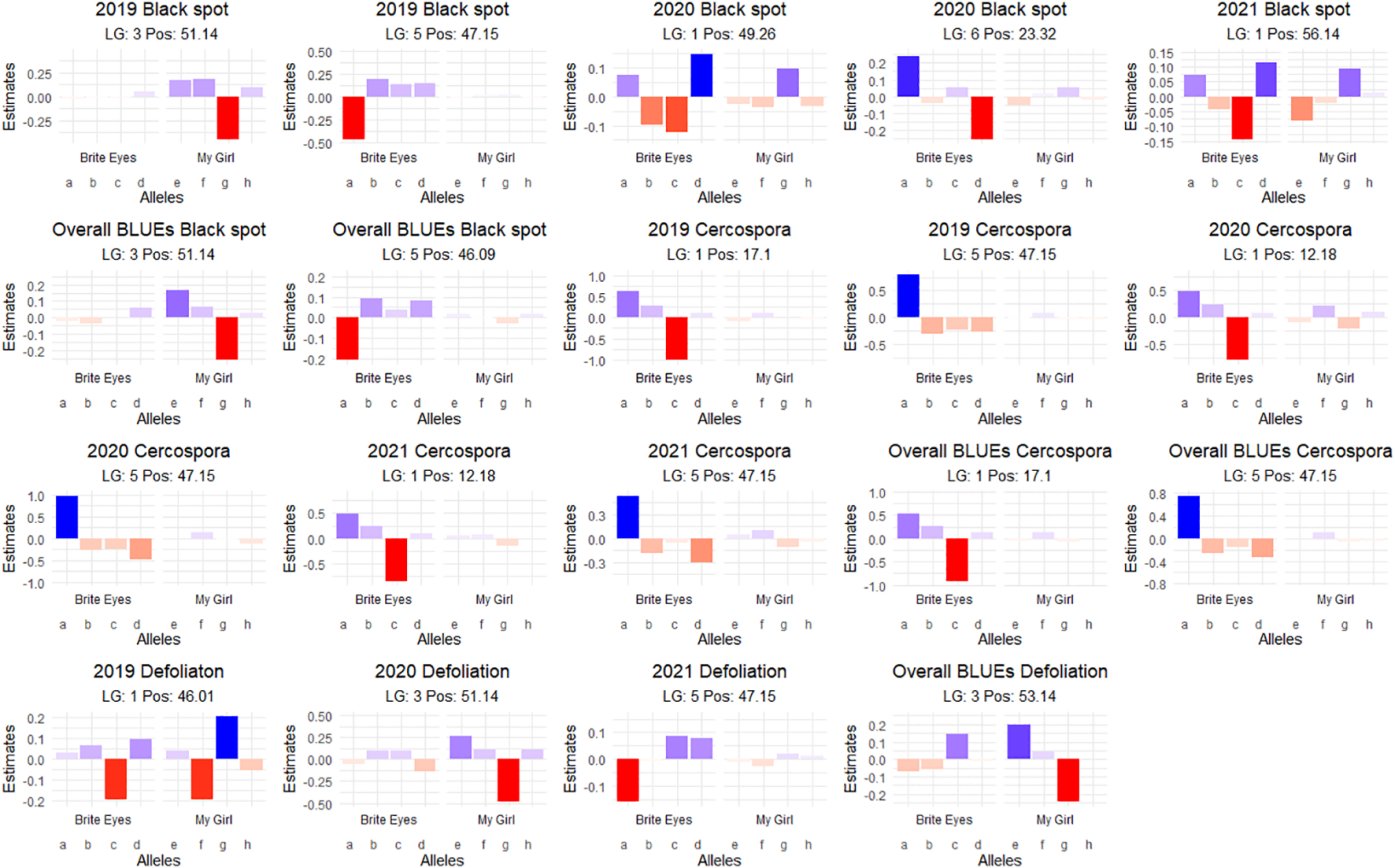
Additive quantitative trait locus (QTL) allele effects of the presence of the parental homolog at the QTL peak on the phenotypic mean measured in the bi-parental garden rose mapping population Brite Eyes × My Girl phenotyped in Somerville, TX, over 3 years.

**Figure 7:**
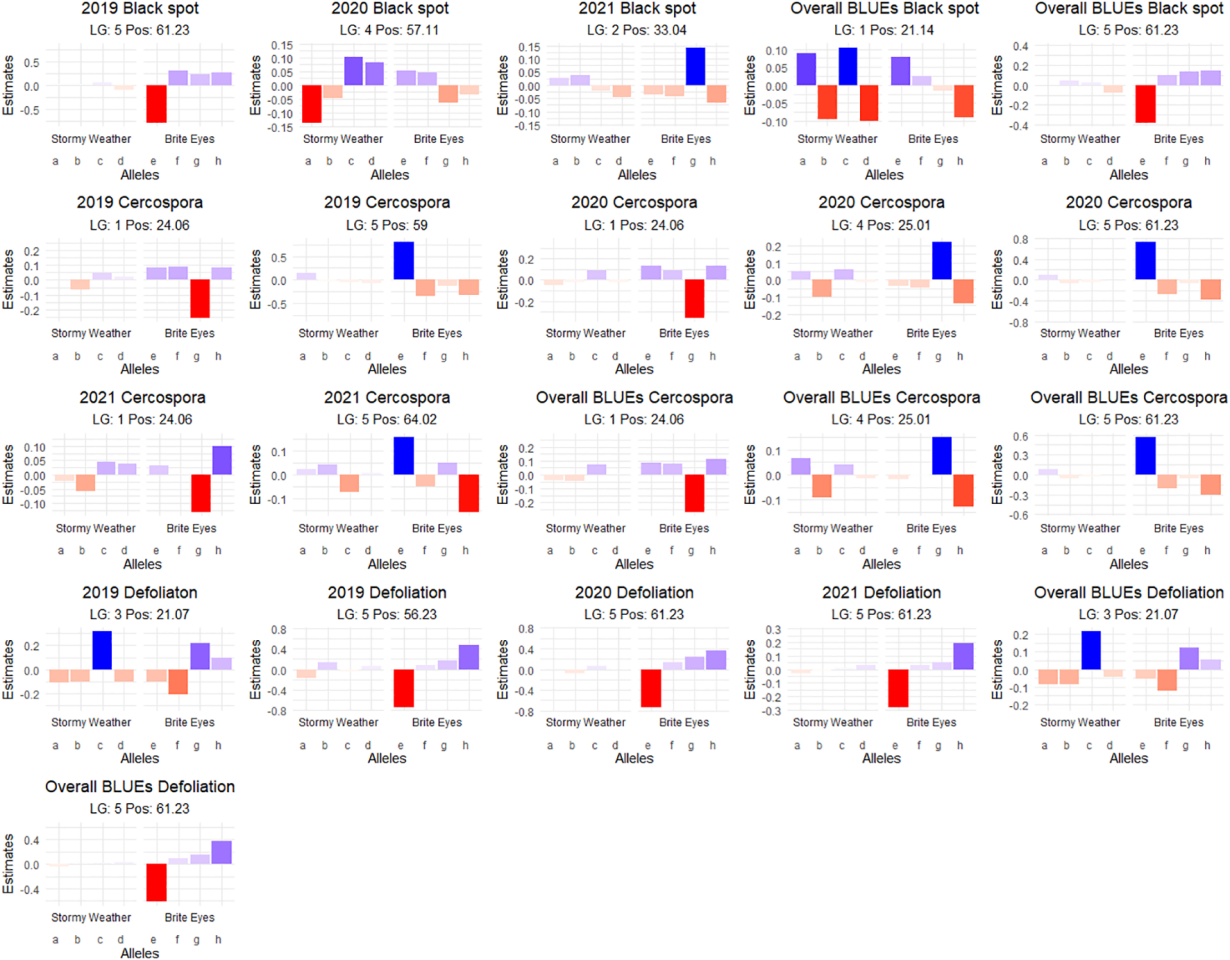
Additive quantitative trait locus (QTL) allele effects of the presence of the parental homolog at the QTL peak on the phenotypic mean measured in the bi-parental garden rose mapping population Stormy Weather × Brite Eyes phenotyped in Somerville, TX, over 3 years.

QTL analysis was performed using linkage maps constructed in a previous study on Rose Rosette resistance utilizing the same populations (Lau et al., 2022). We recognize that all newly discovered QTLs are “putative”, but we will refrain from using this term to avoid confusion with the term “putative QTL” used in QTLpoly. In QTLpoly, a “putative QTL” refers to QTL signals that are close to significance thresholds. QTLs for black spot resistance, cercospora resistance, and defoliation were detected using BLUEs for each genotype estimated from linear mixed models. BLUEs were calculated for each month the data were taken, each year, and overall, for all observations together. Even though QTL scans were conducted for all months separately (**Supplementary Figures 2**, **3**; **Supplementary Tables 1**, **2**, interactive shiny app displaying supplementary data https://github.com/jeekinlau/viewpoly_tetraploid_rose_fungal_disease_supplementary_data), for simplicity, we present QTLs detected using BLUEs calculated from each year and across all years (**Figures 4**–**7**).

We detected QTLs for black spot resistance in the BExMG population on LGs 1, 3, 5, and 6, accounting for 14%–40% of the phenotypic variance explained (PVE), and in the SWxBE population on LGs 1, 2, 4, and 5 accounting for 12%–73% of the PVE (**Tables 2**, **3**). QTLs were detected for cercospora resistance in the BExMG population on LGs 1 and 5 accounting for 19%–43% of the PVE and in the SWxBE population on LGs 1, 4, and 5, accounting for 7%–58% of the PVE. Defoliation QTLs were detected in the BExMG population on LGs 1, 3, and 5, with PVE of 21%–34%, and in the SWxBE population on LGs 3 and 5 with PVE of 11%–56%. Most of the QTLs detected in the bi-parental populations individually were also detected using the diaQTL joint approach (16/19 from BExMG and 16/21 from SWxBE) (**Tables 2**, **3**).

**Table 2:** QTLs detected for black spot and cercospora resistance/susceptibility and defoliation in tetraploid garden rose mapping population Brite Eyes × My Girl phenotyped over 3 years in Somerville, TX.

QTL^a^	Donor parent^b^	LG	LOP^c^	Position (cM)^d^	Position (Mb)^e^	PVE (%)^f^	diaQTL^g^
qRDR.BExMG.2019-ch3	MG ↓	3	9.56	51.14 (44.03–51.14)	38.1 (35.31–40.85)	33	37.73
qRDR.BExMG.2019-ch5	BE ↓	5	>15.65	47.15 (47.15–47.15)	38.31 (38.31–38.31)	35	40.79
qRDR.BExMG.2020-ch1	?	1	3.79	49.26 (2.17–62.07)	54.86 (0.23–64.65)	14	33.87
qRDR.BExMG.2020-ch6	BE ↓	6	4.04	23.32 (17.03–34.24)	17.68 (10.9–38.93)	25	ND
qRDR.BExMG.2021-ch1	?	1	0.37	56.14 (0–69.25)	57.85 (0.23–64.71)	26	ND
qRDR.BExMG.overall-ch3	MG ↓	3	9.54	51.14 (50.28–51.14)	38.1 (38.1–39.43)	40	42.25
qRDR.BExMG.overall-ch5	BE ↓	5	8.00	46.09 (43.1–48.03)	41.23 (29.81–52.44)	24	40.56
qCERC.BExMG.2019-ch1	BE ↓	1	>15.65	17.1 (8.22–22.05)	17.76 (4.9–31.44)	43	27.71
qCERC.BExMG.2019-ch5	BE ↑	5	8.28	47.15 (47.15–48.03)	38.31 (36.38–52.44)	25	39.34
qCERC.BExMG.2020-ch1	BE ↓	1	9.20	12.18 (11.36–20.06)	14.91 (12.5–27.29)	29	22.91
qCERC.BExMG.2020-ch5	BE ↑	5	>15.65	47.15 (47.15–47.15)	38.31 (38.31–38.31)	37	40.04
qCERC.BExMG.2021-ch1	BE ↓	1	9.90	12.18 (8.22–22.05)	14.91 (4.9–31.44)	40	21.76
qCERC.BExMG.2021-ch5	BE ↑	5	5.35	47.15 (44.14–48.03)	38.31 (32.55–52.44)	19	39.84
qCERC.BExMG.overall-ch1	BE ↓	1	>15.65	17.1 (8.22–22.05)	17.76 (4.9–31.44)	40	21.79
qCERC.BExMG.overall-ch5	BE ↑	5	9.11	47.15 (47.15–48.03)	38.31 (36.38–52.44)	27	40.04
qDEF.BExMG.2019-ch1	?	1	0.37	46.01 (0–69.25)	53.23 (0.23–64.71)	21	ND
qDEF.BExMG.2020-ch3	MG ↓	3	6.01	51.14 (39.23–57.05)	38.1 (33.53–45.81)	34	40.92
qDEF.BExMG.2021-ch5	BE ↓	5	3.73	47.15 (24.12–53.02)	38.31 (16–59.29)	23	37.17
qDEF.BExMG.overall-ch3	MG ↓	3	3.79	53.14 (43.02–64.2)	40.29 (35.31–46.74)	30	40.97

a Name of quantitative trait locus (QTL) following naming conventions of the Genome Database for Rosacaceae. RDR, black spot resistance/susceptibility; DEF, defoliation; CERC, cercospora leaf spot resistance/susceptibility.

b Parent contributing allele affecting the phenotypic mean. Estimated by using “qtl_effects” function in QTLpoly. Alleles affecting trait means are followed by the estimated mode of inheritance of the QTL in parentheses and also indicative of whether the allele caused an increase (↑) or decrease (↓) of the mean of the phenotype. In cases with “?”, we were not able to determine any major allele that contributed to the susceptibility or resistance of the trait.

c LOP calculated as −log(p-value) of the QTL peak. p-Values smaller than 2.2e−16 are represented as LOP > 15.65. This is due to limitations of R storing floating point numbers.

d QTL peak position followed by 1.5 LOP confidence intervals in parentheses.

e Physical positions of markers within the 1.5 LOP confidence intervals. WagRhSNP 68k Axiom SNP array probes were aligned to the rose genome assembly by Hibrand-Saint Oyant et al., 2018.

f Percent variance explained (PVE) is estimated in QTLpoly as the QTL heritability ( $h_{q}^{2}$ ) as the ratio of between the variance attributed to the QTL and the total variance.

g Physical position in megabase pairs of QTL peak in joint family analysis using software diaQTL. ND stands for not detected.

**Table 3:** QTLs detected for black spot and cercospora resistance/susceptibility and defoliation in tetraploid garden rose mapping population Stormy Weather × Brite Eyes phenotyped over 3 years in Somerville, TX.

QTL^a^	Donor parent^b^	LG	LOP^c^	Position (cM)^d^	Position (Mb)^e^	PVE (%)^f^	diaQTL^g^
qRDR.SWxBE.2019-ch5	BE ↓	5	>15.65	61.23 (38.06–72.36)	42.58 (16.06–70.92)	73	40.79
qRDR.SWxBE.2020-ch4	SW ↓	4	3.82	57.11 (56.16–64.07)	52.38 (22.32–58.8)	12	ND
qRDR.SWxBE.2021-ch2	BE ↑	2	3.80	33.04 (28.12–39.05)	24.28 (12.19–49.84)	18	ND
qRDR.SWxBE.overall-ch1	?	1	3.75	21.14 (9.11–40.04)	27.59 (8.67–59.76)	15	ND
qRDR.SWxBE.overall-ch5	BE ↓	5	>15.65	61.23 (55.41–68.01)	42.58 (32.69–57.86)	50	40.56
qCERC.SWxBE.2019-ch1	BE ↓	1	5.32	24.06 (21.14–25.23)	30.33 (27.59–38.75)	8	27.71
qCERC.SWxBE.2019-ch5	BE ↑	5	>15.65	59 (49.1–70.06)	38.59 (24.32–60.26)	58	39.34
qCERC.SWxBE.2020-ch1	BE ↓	1	6.76	24.06 (23.31–24.06)	30.33 (28.78–38.75)	13	22.91
qCERC.SWxBE.2020-ch4	BE ↑	4	3.52	25.01 (10.98–30.04)	14.82 (0.64–29.98)	7	ND
qCERC.SWxBE.2020-ch5	BE ↑	5	>15.65	61.23 (49.1–69.27)	42.58 (24.32–58.41)	48	40.04
qCERC.SWxBE.2021-ch1	BE ↓	1	4.06	24.06 (19.06–27.04)	30.33 (21.32–41.49)	12	21.76
qCERC.SWxBE.2021-ch5	BE ↑	5	4.56	64.02 (55.41–72.36)	44.61 (32.69–70.92)	18	39.84
qCERC.SWxBE.overall-ch1	BE ↓	1	7.65	24.06 (23.31–24.06)	30.33 (28.78–38.75)	13	21.79
qCERC.SWxBE.overall-ch4	BE ↑	4	3.70	25.01 (17.11–40.01)	14.82 (0.64–41.85)	7	ND
qCERC.SWxBE.overall-ch5	BE ↑	5	>15.65	61.23 (50.05–69.27)	42.58 (24.61–58.41)	47	40.04
qDEF.SWxBE.2019-ch3	SW ↑	3	5.37	21.07 (19.28–29.15)	27.79 (24.72–34.68)	14	31.43
qDEF.SWxBE.2019-ch5	BE ↓	5	>15.65	56.23 (55.41–63.11)	38.28 (32.69–51.74)	42	36.24
qDEF.SWxBE.2020-ch5	BE ↓	5	>15.65	61.23 (55.41–69.27)	42.58 (32.69–58.41)	56	3.84
qDEF.SWxBE.2021-ch5	BE ↓	5	>15.65	61.23 (60.05–61.23)	42.58 (35.79–51.74)	43	37.17
qDEF.SWxBE.overall-ch3	SW ↑	3	5.16	21.07 (11.06–25.01)	27.79 (8.79–32.89)	11	40.97
qDEF.SWxBE.overall-ch5	BE ↓	5	>15.65	61.23 (48.04–67.08)	42.58 (23.58–57.86)	53	35.83

a Name of quantitative trait locus (QTL) following naming conventions of the Genome Database for Rosacaceae. RDR, black spot resistance/susceptibility; DEF, defoliation; CERC, cercospora leafspot resistance/susceptibility.

b Parent contributing allele affecting the phenotypic mean. Estimated by using “qtl_effects” function in QTLpoly. Alleles affecting trait means are followed by the estimated mode of inheritance of the QTL in parentheses and also indicative of whether the allele caused an increase (↑) or decrease (↓) of the mean of the phenotype. In cases with “?”, we were not able to determine any major allele that contributed to the susceptibility or resistance of the trait.

c LOP calculated as −log(p-value) of the QTL peak. p-Values smaller than 2.2e−16 are represented as LOP > 15.65. This is due to limitations of R storing floating point numbers.

d QTL peak position followed by 1.5 LOP confidence intervals in parentheses.

e Physical positions of markers within the 1.5 LOP confidence intervals. WagRhSNP 68k Axiom SNP array probes were aligned to the rose genome assembly by Hibrand-Saint Oyant et al., 2018.

f Percent variance explained (PVE) is estimated in QTLpoly as the QTL heritability ( $h_{q}^{2}$ ) as the ratio of between the variance attributed to the QTL and the total variance.

g Physical position in megabase pairs of QTL peak in joint family analysis using software diaQTL. ND stands for not detected.

## 4 Discussion

As discussed in the Introduction, many factors make it difficult for studying black spot disease in a landscape-like environment. However, we used multiple time points over the course of 3 years to look at the temporal progression of the disease to help overcome the uncertainty of weather conditions. Using a mixed model approach, we were able to estimate the BLUEs for each genotype that best represents the performance of each genotype while taking into account the environmental variance. A major QTL for black spot resistance was detected on LG 5 in 2019 and in the overall analysis. This QTL was localized to a region on LG 5 where the black spot resistance locus, *Rdr4*, was previously mapped (Zurn et al., 2018) (**Figure 5**). Thus, our black spot resistance QTL on LG 5 likely represents the *Rdr4* locus since the favorable black spot resistance QTL allele, in our study, came from Brite Eyes, the cultivar originally used to localize *Rdr4* genetically (Zurn et al., 2018). The location of the *Rdr4* locus on LG 5 was first determined using detached leaf assays in a Brite Eyes × Morden Blush mapping population of 94 individuals (Zurn et al., 2018). Our study complements this previous work and provides additional information to better localize the *Rdr4* locus on LG 5 since we used QTL mapping methods with roughly twice the number of individuals (n = 157 and n = 200) phenotyped over the course of 3 years. The black spot meta-QTL study reported by Lopez Arias et al. (2020) posited that the two meta-QTLs found on LG 5 were either two novel QTLs not associated with *Rdr4* or that Meta_2_5 was the same genetic factor as *Rdr4*, but their locations did not collocate due to mapping imprecisions. The confidence intervals of our LG 5 QTLs, qRDR.BExMG.2019-ch5, qRDR.BExMG.overall-ch5, qRDR.SWxBE.2019-ch5, and qRDR.SWxBE.overall-ch5, are near but not collinear with the estimated position of Meta_2_5. In contrast, our LG 5 QTL for black spot resistance coincides with the reported position of *Rdr4* (the confidence intervals overlap with the reported position of *Rdr4* with our QTL peaks being a bit downstream of its reported location). Since the meta-QTL studies of Lopez Arias et al. (2020) used diploid germplasm unrelated to our populations and we found the coincidence of our LG5 QTL and *Rdr4*, our findings indicate that Meta_2_5 may be another genetic factor controlling black spot resistance further upstream of *Rdr4*. A gene search in the *Rosa chinensis* whole genome v1.0 assembly (Hibrand-Saint Oyant et al., 2018) in the region where we mapped black spot resistance QTL coinciding with the location of *Rdr4* (29.81–57.86 Mbp) revealed the presence of eight TIR-NBS-LRR-, one CC-NBS-LRR-, and 26 NB-ARC-related genes, all of which are related to general disease resistance mechanisms (**Supplementary Material 1**). In addition to resistance genes, there were seven cutin, suberin, and wax biosynthesis-related genes. A thicker coat of cutin or wax on the leaf surfaces and around the plant cells can create a physical barrier against fungal pathogen attacks (Arya et al., 2021). There were also two ACD6 ankyrin repeat family proteins, which are activators of a salicylic acid pathway and involved in accelerated cell death, which is a defense response against viral, bacterial, and fungal pathogens (Lu et al., 2003). There was one MLP-like protein, Bet v-1, a defense response protein in response to biotic stimuli (Chen and Dai, 2010). Although the coincidence of disease resistance genes and our QTL in LG 5 (*Rdr4*) is interesting, additional work is needed to better localize our QTL and to identify more suitable candidate genes in the pertinent germplasm.

Even though we detected a strong QTL that we believe represents the *Rdr4* locus in the overall analysis, we find that the resistance imparted by this locus waned in 2020 and 2021. This locus was still detected in data in May 2020 in both populations and in May 2021 in the SWxBE population (**Supplementary Figures 2**, **3**), but this QTL was undetected in later months of 2020 and 2021 or in yearly analyses. The loss of this QTL is visualized by a shift in the distribution of black spot resistance scores (**Figure 2**) where resistant genotypes present in 2019 were no longer resistant in subsequent years. Assuming that *Rdr4* underlies our QTL on LG 5, explanations for the loss of resistance include the evolution of a strain(s) able to overcome the *Rdr4* in our location or 2) the selection of black spot strain(s) able to overcome *Rdr4* that were initially present in low frequency. One of the potential limitations of this study is the absence of a host differential set (Whitaker et al., 2007; Zlesak et al., 2020). The inclusion of a host differential set may have given us insight into the races we may have in the field and may have helped us better understand this shift in resistance/susceptibility. However, it is worth noting that the host differential sets are scored using detached leaf assays; thus, it may not be directly transferable to a field-based experiment.

The other major QTLs for black spot resistance were on LG 3 and were detected in the 2019 and overall QTL scans. The LG 3 QTLs (qRDR.BExMG.2019-ch3 and qRDR.BExMG.overall-ch3) from My Girl collocate with Meta_2_3 (Lopez Arias et al., 2020) and overlapped with the qBSD.TX2WSE-LG3.4 QTLs (Rawandoozi et al., 2022) that span the same area as Meta_2_3. Therefore, our QTL may be the same genetic factor detected in both the meta-analysis and the joint family QTL analysis on diploid germplasm. Upon closer inspection of the monthly QTL scans (**Supplementary Figure 2**), we also see that this resistance QTL is also not present in the years 2020 and 2021. Like qBSD.TX2WSE-LG3.4, our LG 3 QTL for black spot resistance only shows up in the first year and the overall analysis. The region of the black spot resistance QTL on LG 3 is between 35.31 and 40.85 Mbp. No disease resistance-related genes were found in this region.

Interestingly, there is a cercospora leaf spot susceptibility QTL allele that colocalized with the black spot resistance QTL on LG 5 (*Rdr4*). The QTL is inherited from the same parental homolog (**Figures 6**, **7**). This QTL spans 24.61–58.41 Mbp, and there are nine TIR-NBS-LRR-, one CC-NBS-LRR-, 26 NB-ARC-related genes and one predicted resistance protein in the region (**Supplementary Material 2**). There were also seven cutin, suberine, and wax biosynthesis-related genes; one MLO11 transmembrane gene; four Bet v 1 MLP defense response genes; and two ACD6 accelerated cell death genes. Unlike the black spot QTL representing *Rdr4*, the colocalizing cercospora susceptibility QTL on LG 5 was stable across time. Thus, the two possibilities are that two tightly linked genetic factors control these two traits with opposing effects, or the same underlying genetic factor provided both resistance to black spot and susceptibility to cercospora leaf spot. From a plant breeder’s perspective, one would like to break this linkage (if two genetic factors were present) to create progeny that have black spot resistance without cercospora susceptibility. However, if there was only one genetic factor controlling these two traits, this association cannot be broken. In either case, further investigation will require the creation of larger mapping populations to capture more recombination breakpoints to determine if there are one or two genetic factors.

**Figure 8:**
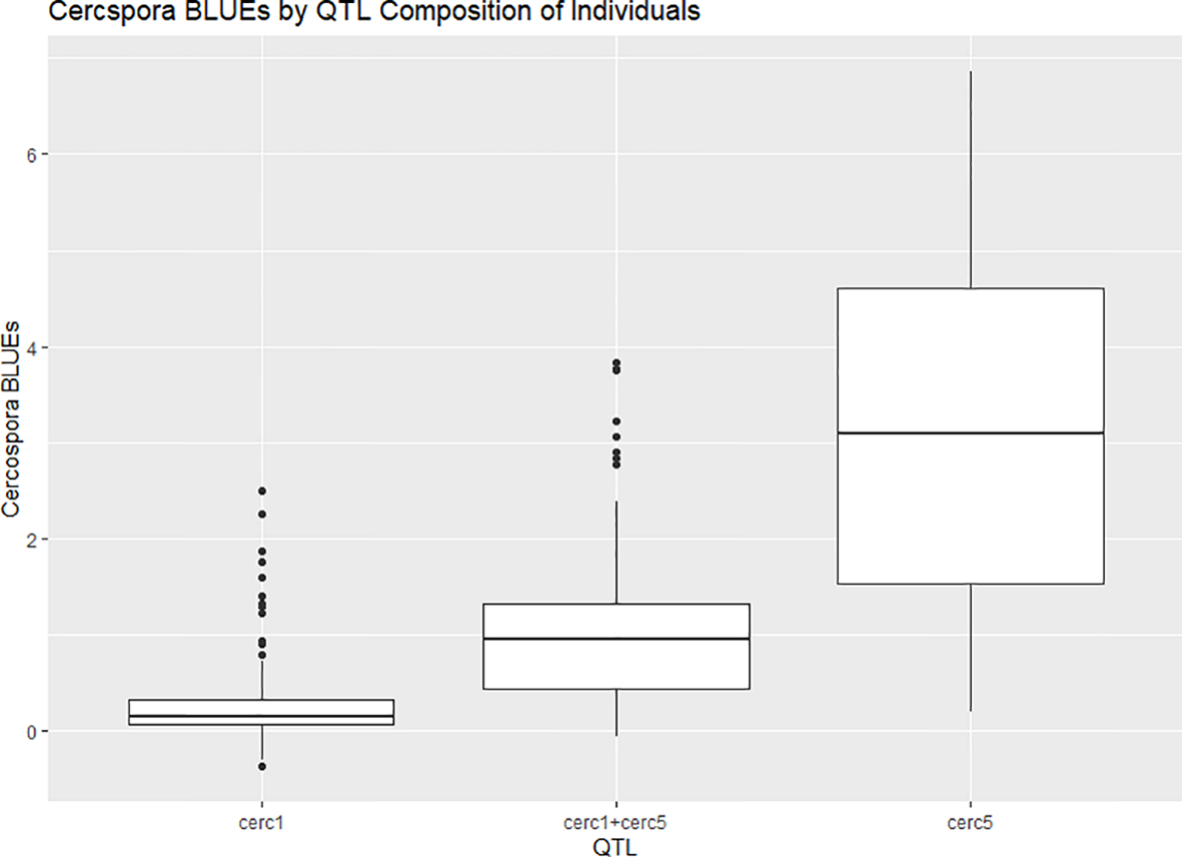
Boxplots of phenotypic best linear unbiased estimates (BLUEs) of cercospora disease ratings phenotyped in two bi-parental mapping populations of tetraploid garden rose phenotyped in Somerville, TX, over the course of 3 years, grouped by quantitative trait locus (QTL) composition.

A major QTL for cercospora resistance was also detected on LG 1 in both populations where a simplex factor from Brite Eyes lowered cercospora disease incidence. The QTL region on LG 1 spans 4.9–38.75 Mbp and has 56 TIR-NBS-LRR-, 11 CC-NBS-LRR-, 64 NB-ARC-related genes within this region along with several predicted disease resistance genes (**Supplementary Material 3**). In addition to these general resistance genes are three Fe superoxide dismutase-related genes; one cutin, suberine, and wax biosynthesis-related gene; two MLP-related genes; and two MLO-related genes. This cercospora QTL on LG 1 is not linked with any black spot resistance or susceptibility QTL and was also stable over time. With respect to breeding for resistance, it may be desirable to stack the black spot resistance factor on LG 5 (*Rdr4*) with the cercospora resistance factor on LG 1. The BExMG population has 34 individuals, and the SWxBE population has 53 individuals with both of these resistance factors. In the case of the BExMG population, we can also select individuals carrying black spot resistance factors in LG 3 and LG 5 as well as the LG 1 cercospora resistance factor (n = 22). The recent progress in developing tools for polyploids allows the rapid characterization of individuals with respect to QTL composition, which, in turn, enables the selection of genotypes that carry multiple favorable alleles for their use in subsequent breeding. One could argue that since the black spot resistance QTL on LG 5 (Rdr4) has lost effectiveness over time while the QTL for cercospora susceptibility on LG 5 is stable over time, one should avoid using black spot resistance in LG 5 in order to avoid susceptibility to cercospora leaf spot; however, while that may be the case for our specific environment, in other less humid and cooler environments (where cercospora is less of a problem), it may beneficial to have the *Rdr4* black spot resistance locus. Depending on the target environment, one may choose one trait over the other. However, for our environment, having the cercospora resistance QTL on LG 1 may be more important than selecting against the cercospora susceptible QTL on LG 5. Our analysis shows that having both cercospora resistance on LG1 and cercospora leaf spot susceptibility QTL on LG5 is not as deleterious as only selecting for the susceptibility factor on LG 5 (**Figure 8**). Thus, if one selects for *Rdr4* (and consequently selects for cercospora susceptibility on LG 5), cercospora resistance on LG 1 should also be selected.

Defoliation QTLs were not detected consistently every year, with the exception of the QTL on LG 5 in the SWxBE population. Defoliation is caused by both biotic and abiotic factors; thus, inconsistent detection of QTL from year to year is not surprising. Black spot and defoliation are moderately correlated with each other (**Figure 2**), and as expected, there was some collinearity between black spot resistance and defoliation QTLs on LGs 3 and 5 in the BExMG population and on LG 5 in the SWxBE population. Even though both mapping populations had defoliation QTL on LG 3, the QTL from SWxBE is inherited from Stormy Weather between 8.79 and 34.68 Mbp, and in the BExMG population, it is inherited from My Girl between 33.53 and 46.74 Mbp. The defoliation QTL on LG 3 collocated with the blackspot QTL on LG 3 only in the BExMG population, and the two defoliation QTLs appear to be separate QTLs due to the physical location of the QTLs. Because black spot infections result in defoliation, we expected to see some overlap between black spot resistance and defoliation QTL; however, there are other biotic and abiotic factors that also contribute to defoliation. Thus, it is plausible that defoliation QTLs do not collocate with any black spot resistance QTL due to other factors (defoliation QTL on LG 3 in the SWxBE population) or diseases (defoliation QTL on LG 1 in the BExMG population is near the cercospora resistance QTL on LG 1). The defoliation QTL on LG 3 in the SWxBE population from 8.79 to 32.89 Mbp contained 10 heat shock-related proteins, which play a key role in plants when plants are exposed to heat stress (Kotak et al., 2007). A few other general stress response genes like AN1-like Zinc fingers and genes with peroxidase functions were also found (**Supplementary Material 4**).

The goal of studying our mapping populations was to gain insight into the genetic architecture of disease resistance and begin pyramiding resistance alleles within the context of relevant germplasm. Newly developed software tools allowed us to quickly identify individuals that carry multiple alleles of interest for use in breeding. For example, in the BExMG population, one may want to select for resistance to black spot on LG 3 and 5, cercospora resistance on LG 1, and RRD resistance previously discovered on LG 5 (Lau et al., 2022). The Shiny app VIEWpoly allows the identification of individuals with the desired homologs and displays progeny with all four QTLs along with the homolog probabilities at each QTL interval (**Supplementary Figure 4**). Assuming a naïve independent assortment of QTL, the odds of recovering any QTL from these four simplex QTL are 50% when crossing with individuals with no QTL (nulliplex). Thus, we should expect 0.5^4^ or 6.25% of individuals to have one copy of all four resistance QTLs. Interestingly, a cross between two of these elite full sibs that already carry all four QTLs should yield progeny where for each QTL, 75% of the progeny carry at least one copy and 25% of them will carry two copies of the QTL in question (simplex-by-simplex segregation ratios are 1 duplex : 2 simplex : 1 nulliplex). Assuming independent segregation, 0.75^4^ = 31.64% of the progeny in the crosses with all four QTLs would have at least all four QTLs in simplex configuration, and 0.25^4^ = 0.39% would have all four QTLs containing two copies of the favorable allele. Tetraploid roses can tolerate some level of inbreeding; thus, intercrossing progeny identified to carry resistance QTLs for all four traits may be a viable breeding strategy. Using these discovered QTLs, we can begin to stack alleles of interest to improve disease resistance, and combined with other QTLs for aesthetic traits like low defoliation or high flowering intensity, we can create genotypes that have favorable alleles for multiple traits. However, further work needs to be performed to develop user-friendly diagnostics to track the inheritance of the QTL.

## 5 Conclusion

In conclusion, we used two interconnected mapping populations to study the inheritance of resistance to black spot and cercospora leaf spot as well as defoliation. We discovered a black spot resistance QTL on LG 3 that may be the same factor as Meta_2_3 reported in diploid studies. Another black spot resistance QTL on LG 5 is believed to be the *Rdr4* black spot resistance locus. We discovered a novel cercospora leaf spot resistance QTL on LG 1 and a cercospora susceptibility QTL on LG 5. However, due to the linkage between black spot resistance and cercospora susceptibility QTL on LG 5, caution is needed to use these for fungal disease resistance breeding. Defoliation QTLs were described for the first time, two of which coincided with black spot resistance QTL, one coincided with a cercospora resistance QTL, and one that did not collocate with either of the diseases. The mapping populations used in this research are currently maintained to study other traits of interest and will continue to add to the body of knowledge on tetraploid garden roses and polyploid genetics.

## Data availability statement

The data presented in the study are deposited in the Genome Database for Rosaceae repository (https://www.rosaceae.org/publication_datasets), accession number tfGDR1068.

## Author contributions

DB conceived the experiment. JL, OR-L, and DB prepared and edited the manuscript, and JL and EY propagated and established populations in the field. JL and HG took the phenotypic data. PK provided funding for DNA extraction. JL performed phenotypic data statistics, linkage maps, and QTL mapping. JL, DB, and OR-L worked on the interpretation of data. All authors contributed to the article and approved the submitted version.

## Supplementary material

Supplementary Figure 1 Correlations between black spot **(B)**, cercospora **(C)**, and defoliation **(D)** yearly and overall phenotypic BLUEs phenotyped in two bi-parental mapping populations of tetraploid garden rose phenotyped in Somerville, TX, over the course of three years.

Supplementary Figure 2 QTL scans for **(A)** black spot, **(B)** cercospora, and **(C)** defoliation detected in autotetraploid garden rose bi-parental mapping population, Brite Eyes x My Girl. Year followed by number denotes QTL scan on a particular month (for example 2019_6 denotes June 2019). Year followed by BLUEs denotes QTL scan on yearly BLUEs. Overall_BLUEs denotes BLUEs calculated from all months together.

Supplementary Figure 3 QTL scans for **(A)** black spot, **(B)** cercospora, and **(C)** defoliation detected in autotetraploid garden rose bi-parental mapping population, Stormy Weather x Brite Eyes. Year followed by number denotes QTL scan on a particular month (for example 2019_6 denotes June 2019). Year followed by BLUEs denotes QTL scan on yearly BLUEs. Overall_BLUEs denotes BLUEs calculated from all months together.

Supplementary Figure 4 Screenshot of R Shiny app VIEWpoly displaying homolog probabilities for progeny with specific parental homologs selected for four different QTL on three chromosomes.

Supplemental File 1 Supplementary Tables 1 and 2, QTL for all black spot, cercospora, and defoliation for the garden rose tetraploid populations Brite Eyes x My Girl and Stormy Weather x Brite Eyes phenotyped over 3 years in Somerville, TX. BLUEs were calculated monthly, yearly, and overall.

Supplemental File 2 Disease resistance related gene search for black spot QTL on LG 5.

Supplemental File 3 Disease resistance related gene search for cercospora QTL on LG 5.

Supplemental File 4 Disease resistance related gene search for cercospora QTL on LG 1.

Supplemental File 5 Heat and drought stress related gene search for defoliation QTL on LG 3.
