# Supplementary figures and images for: QTL discovery for resistance to black spot and cercospora leaf spot, and defoliation in two interconnected F1 bi-parental tetraploid garden rose populations
Source: Front Plant Sci. 2023 Jul 27;14:1209445. doi: 10.3389/fpls.2023.1209445 (PMC10413565; doi:10.3389/fpls.2023.1209445)

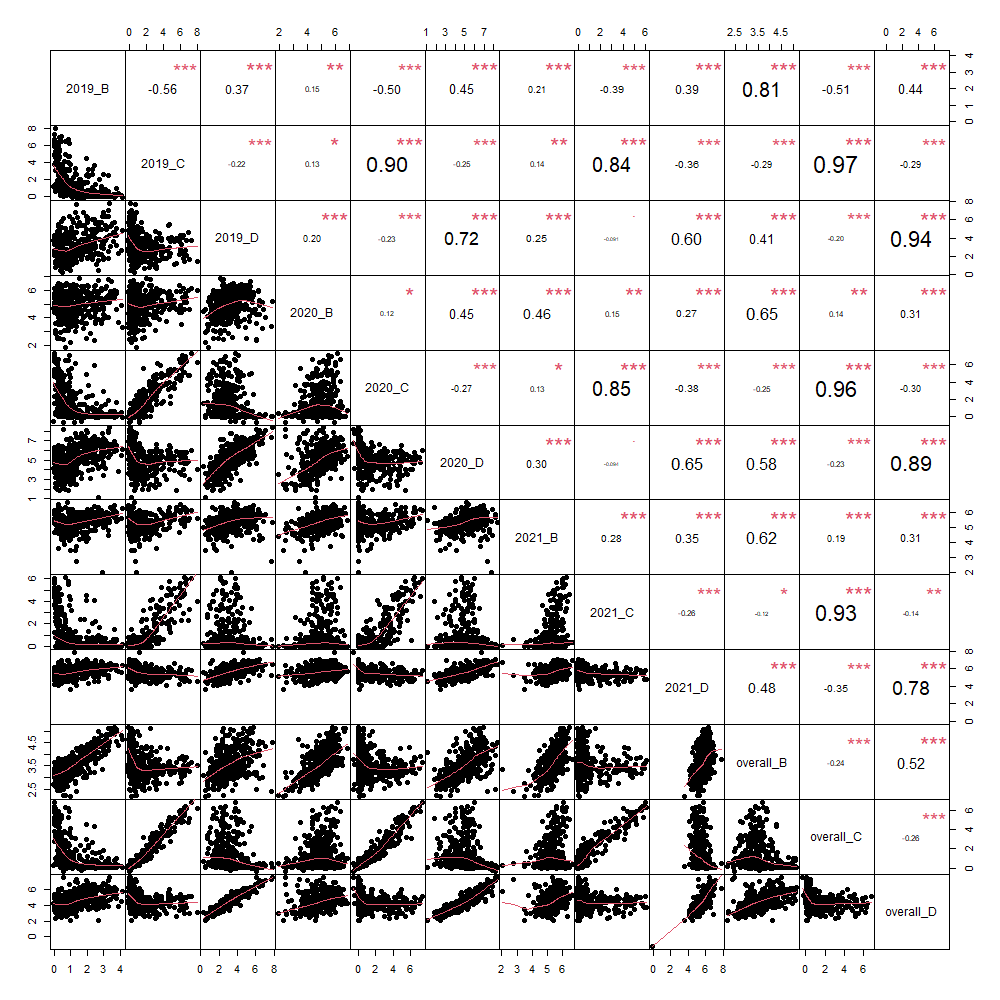

Supplement: Supplementary Figure 1 — Correlations between black spot (B), cercospora (C), and defoliation (D) yearly and overall phenotypic BLUEs phenotyped in two bi-parental mapping populations of tetraploid garden rose phenotyped in Somerville, TX, over the course of three years. [file Image_1.tiff]

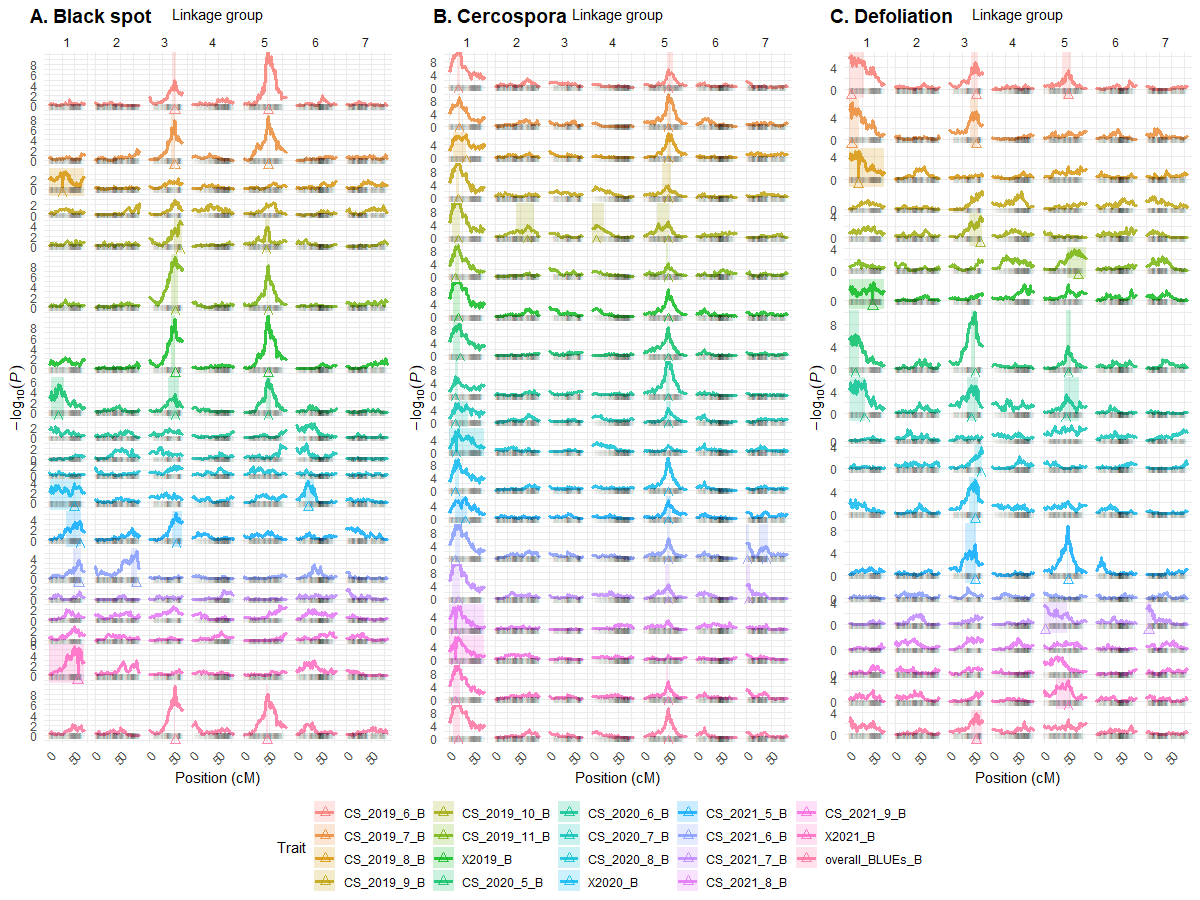

Supplement: Supplementary Figure 2 — QTL scans for (A) black spot, (B) cercospora, and (C) defoliation detected in autotetraploid garden rose bi-parental mapping population, Brite Eyes x My Girl. Year followed by number denotes QTL scan on a particular month (for example 2019_6 denotes June 2019). Year followed by BLUEs denotes QTL scan on yearly BLUEs. Overall_BLUEs denotes BLUEs calculated from all months together. [file Image_2.tiff]

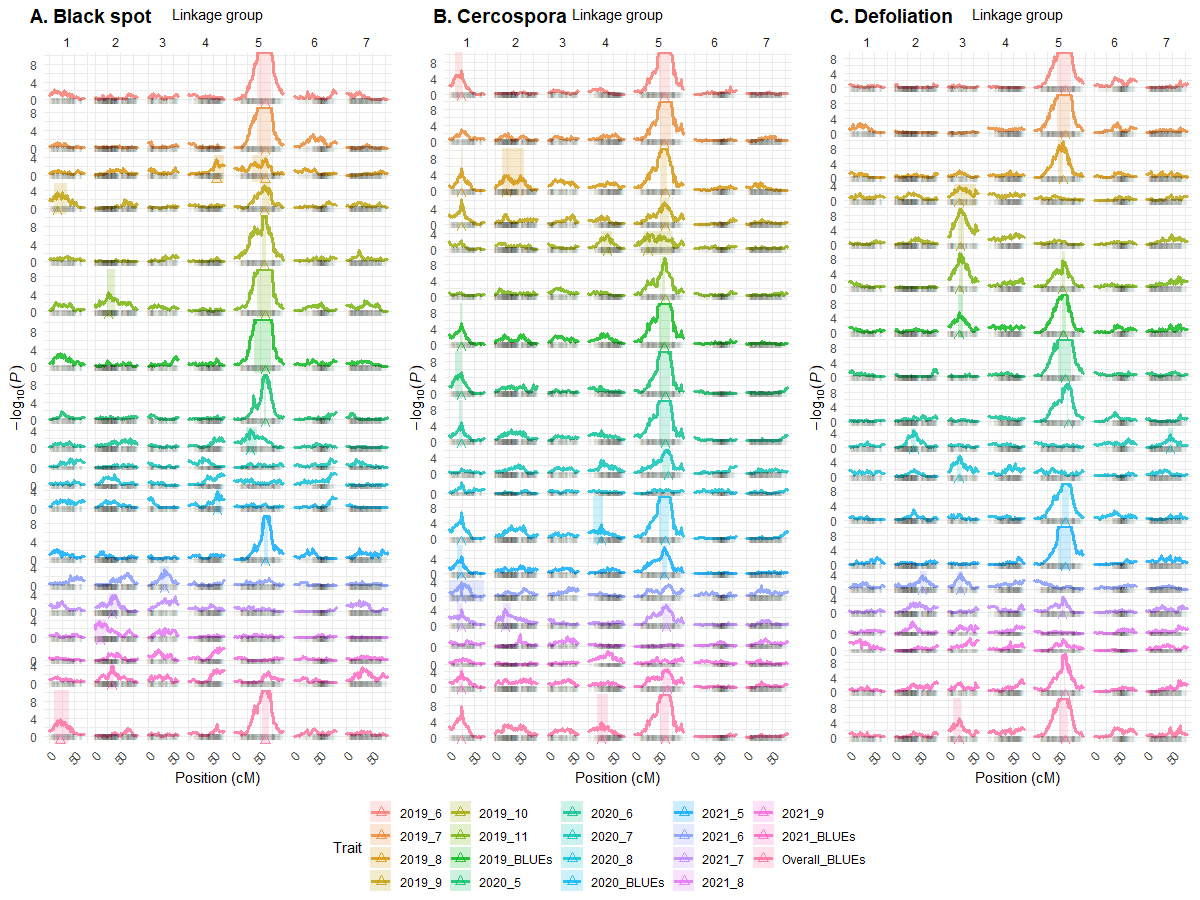

Supplement: Supplementary Figure 3 — QTL scans for (A) black spot, (B) cercospora, and (C) defoliation detected in autotetraploid garden rose bi-parental mapping population, Stormy Weather x Brite Eyes. Year followed by number denotes QTL scan on a particular month (for example 2019_6 denotes June 2019). Year followed by BLUEs denotes QTL scan on yearly BLUEs. Overall_BLUEs denotes BLUEs calculated from all months together. [file Image_3.tiff]

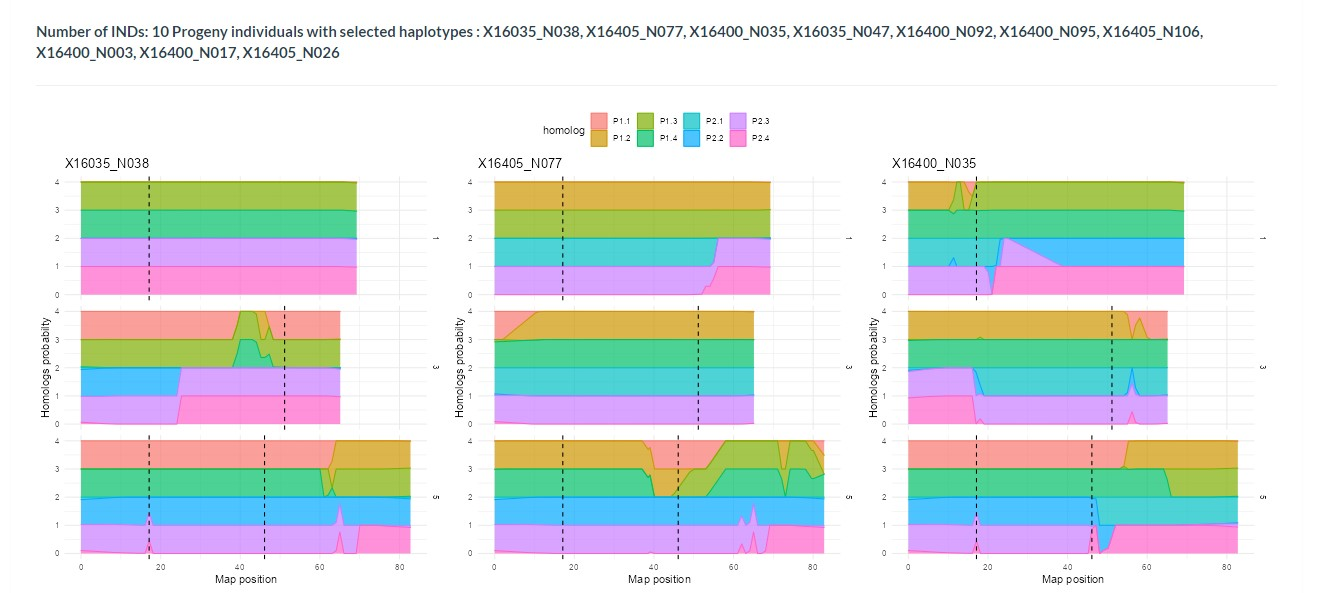

Supplement: Supplementary Figure 4 — Screenshot of R Shiny app VIEWpoly displaying homolog probabilities for progeny with specific parental homologs selected for four different QTL on three chromosomes. [file Image_4.tiff]
