# Supplementary material for: QTL discovery for resistance to black spot and cercospora leaf spot, and defoliation in two interconnected F1 bi-parental tetraploid garden rose populations
Source: Front Plant Sci. 2023 Jul 27;14:1209445. doi: 10.3389/fpls.2023.1209445 (PMC10413565; doi:10.3389/fpls.2023.1209445)
Supplement: Supplemental File 1 — Supplementary Tables 1 and 2, QTL for all black spot, cercospora, and defoliation for the garden rose tetraploid populations Brite Eyes x My Girl and Stormy Weather x Brite Eyes phenotyped over 3 years in Somerville, TX. BLUEs were calculated monthly, yearly, and overall. [file Table_1.docx]

**Supplementary Table 1**. QTL for resistance/tolerance to black spot, cercospora, and defoliation for the tetraploid garden rose population Brite Eyes x My Girl phenotyped over 3 years in Somerville, TX. BLUEs were calculated monthly, yearly, and overall.

| Trait^a^ | | Linkage group | Position (cM) | | Lower interval (cM) | | Upper interval (cM) | | p-value | | h^2 B^ | |  |
| --- | --- | --- | --- | --- | --- | --- | --- | --- | --- | --- | --- | --- | --- |
| 2019_6_B | 3 | | | 50.28 | | 45.21 | | 53.14 | | 1.16E-05 | | 0.18 | |
| 2019_6_B | 5 | | | 47.15 | | 46.09 | | 47.15 | | <2.22e-16 | | 0.45 | |
| 2019_7_B | 3 | | | 50.28 | | 44.03 | | 51.14 | | 1.93E-08 | | 0.32 | |
| 2019_7_B | 5 | | | 47.15 | | 46.09 | | 48.03 | | 3.01E-09 | | 0.3 | |
| 2019_8_B | 1 | | | 26.24 | | 0 | | 69.25 | | 4.27E-01 | | 0.21 | |
| 2019_10_B | 3 | | | 61.03 | | 50.28 | | 65.12 | | 1.23E-05 | | 0.34 | |
| 2019_10_B | 5 | | | 44.14 | | 41.04 | | 49 | | 1.69E-04 | | 0.15 | |
| 2019_11_B | 3 | | | 51.14 | | 44.03 | | 58.11 | | 1.79E-10 | | 0.32 | |
| 2019_11_B | 5 | | | 47.15 | | 46.09 | | 48.03 | | 5.83E-09 | | 0.29 | |
| 2019_B | 3 | | | 51.14 | | 44.03 | | 51.14 | | 2.75E-10 | | 0.33 | |
| 2019_B | 5 | | | 47.15 | | 47.15 | | 47.15 | | <2.22e-16 | | 0.35 | |
| 2020_5_B | 1 | | | 18.32 | | 4.02 | | 30.25 | | 4.53E-06 | | 0.19 | |
| 2020_5_B | 3 | | | 51.14 | | 37.02 | | 60.02 | | 3.69E-04 | | 0.14 | |
| 2020_5_B | 5 | | | 47.15 | | 43.1 | | 54.15 | | 4.68E-07 | | 0.27 | |
| 2020_B | 1 | | | 49.26 | | 2.17 | | 62.07 | | 1.64E-04 | | 0.14 | |
| 2020_B | 6 | | | 23.32 | | 17.03 | | 34.24 | | 9.05E-05 | | 0.25 | |
| 2021_5_B | 1 | | | 61 | | 33.35 | | 63.04 | | 2.25E-04 | | 0.13 | |
| 2021_5_B | 3 | | | 53.14 | | 45.21 | | 64.2 | | 6.91E-06 | | 0.28 | |
| 2021_6_B | 1 | | | 58.02 | | 48.03 | | 62.07 | | 3.07E-04 | | 0.11 | |
| 2021_6_B | 2 | | | 82.02 | | 71.03 | | 84.05 | | 4.29E-06 | | 0.28 | |
| 2021_B | 1 | | | 56.14 | | 0 | | 69.25 | | 4.27E-01 | | 0.26 | |
| overall_BLUEs_B | 3 | | | 51.14 | | 50.28 | | 51.14 | | 2.86E-10 | | 0.4 | |
| overall_BLUEs_B | 5 | | | 46.09 | | 43.1 | | 48.03 | | 1.01E-08 | | 0.24 | |
| 2019_6_C | 1 | | | 17.1 | | 15.12 | | 22.05 | | <2.22e-16 | | 0.41 | |
| 2019_6_C | 5 | | | 47.15 | | 46.09 | | 57 | | 4.69E-06 | | 0.19 | |
| 2019_7_C | 1 | | | 19.05 | | 17.1 | | 22.05 | | 1.49E-09 | | 0.27 | |
| 2019_7_C | 5 | | | 47.15 | | 46.09 | | 53.02 | | 1.71E-10 | | 0.37 | |
| 2019_8_C | 1 | | | 33.35 | | 8.22 | | 33.35 | | 6.55E-08 | | 0.26 | |
| 2019_8_C | 5 | | | 47.15 | | 46.09 | | 54.15 | | 5.09E-08 | | 0.31 | |
| 2019_9_C | 1 | | | 17.1 | | 14.01 | | 20.06 | | <2.22e-16 | | 0.44 | |
| 2019_9_C | | 5 | 47.15 | | 36.26 | | 53.02 | | 1.71E-04 | | 0.14 | |  |
| 2019_10_C | | 1 | 17.1 | | 6.08 | | 21.01 | | <2.22e-16 | | 0.45 | |  |
| 2019_10_C | | 2 | 64.06 | | 43 | | 78.08 | | 2.04E-04 | | 0.08 | |  |
| 2019_10_C | | 4 | 6.99 | | 0 | | 22.2 | | 1.50E-04 | | 0.09 | |  |
| 2019_10_C | | 5 | 47.15 | | 26.23 | | 49 | | 8.71E-06 | | 0.11 | |  |
| 2019_11_C | | 1 | 12.18 | | 11.36 | | 20.06 | | 7.90E-10 | | 0.38 | |  |
| 2019_11_C | | 5 | 47.15 | | 43.1 | | 48.03 | | 8.18E-05 | | 0.16 | |  |
| 2019_C | | 1 | 17.1 | | 8.22 | | 22.05 | | <2.22e-16 | | 0.43 | |  |
| 2019_C | | 5 | 47.15 | | 47.15 | | 48.03 | | 5.26E-09 | | 0.25 | |  |
| 2020_5_C | | 1 | 22.05 | | 8.22 | | 22.05 | | 1.65E-10 | | 0.33 | |  |
| 2020_5_C | | 5 | 47.15 | | 46.09 | | 48.03 | | 2.19E-09 | | 0.29 | |  |
| 2020_6_C | | 1 | 11.36 | | 11.36 | | 17.1 | | 7.55E-07 | | 0.16 | |  |
| 2020_6_C | | 5 | 47.15 | | 44.14 | | 49 | | <2.22e-16 | | 0.53 | |  |
| 2020_7_C | | 1 | 12.18 | | 11.36 | | 38.07 | | 1.41E-06 | | 0.25 | |  |
| 2020_7_C | | 5 | 47.15 | | 44.14 | | 53.02 | | 4.11E-05 | | 0.21 | |  |
| 2020_8_C | | 1 | 13.01 | | 0 | | 69.25 | | 4.28E-01 | | 0.36 | |  |
| 2020_C | | 1 | 12.18 | | 11.36 | | 20.06 | | 6.35E-10 | | 0.29 | |  |
| 2020_C | | 5 | 47.15 | | 47.15 | | 47.15 | | <2.22e-16 | | 0.37 | |  |
| 2021_5_C | | 1 | 32.03 | | 11.36 | | 35.15 | | 3.82E-07 | | 0.3 | |  |
| 2021_5_C | | 5 | 47.15 | | 43.1 | | 53.02 | | 3.29E-06 | | 0.22 | |  |
| 2021_6_C | | 1 | 12.18 | | 11.36 | | 22.05 | | <2.22e-16 | | 0.3 | |  |
| 2021_6_C | | 5 | 48.03 | | 46.09 | | 49 | | 4.99E-07 | | 0.17 | |  |
| 2021_6_C | | 7 | 2.41 | | 0 | | 7.01 | | 1.96E-05 | | 0.16 | |  |
| 2021_6_C | | 7 | 39.18 | | 26.04 | | 43.07 | | 2.32E-04 | | 0.11 | |  |
| 2021_7_C | | 1 | 17.1 | | 6.08 | | 22.05 | | <2.22e-16 | | 0.45 | |  |
| 2021_7_C | | 5 | 47.15 | | 41.04 | | 49 | | 3.42E-05 | | 0.11 | |  |
| 2021_7_C | | 7 | 4.13 | | 0 | | 8.02 | | 4.36E-05 | | 0.12 | |  |
| 2021_8_C | | 1 | 12.18 | | 0 | | 69.25 | | 4.28E-01 | | 0.37 | |  |
| 2021_9_C | | 1 | 12.18 | | 0 | | 69.25 | | 4.28E-01 | | 0.38 | |  |
| 2021_C | | 1 | 12.18 | | 8.22 | | 22.05 | | 1.27E-10 | | 0.4 | |  |
| 2021_C | | 5 | 47.15 | | 44.14 | | 48.03 | | 4.43E-06 | | 0.19 | |  |
| overall_BLUEs_C | | 1 | 17.1 | | 8.22 | | 22.05 | | <2.22e-16 | | 0.4 | |  |
| overall_BLUEs_C | | 5 | 47.15 | | 47.15 | | 48.03 | | 7.80E-10 | | 0.27 | |  |
| 2019_6_D | | 1 | 3.34 | | 0 | | 29.09 | | 6.55E-07 | | 0.24 | |  |
| 2019_6_D | | 3 | 53.14 | | 44.03 | | 57.05 | | 1.51E-05 | | 0.22 | |  |
| 2019_6_D | | 5 | 47.15 | | 35.26 | | 53.02 | | 3.26E-04 | | 0.16 | |  |
| 2019_7_D | | 1 | 5.04 | | 0 | | 20.06 | | 1.86E-07 | | 0.27 | |  |
| 2019_7_D | | 3 | 53.14 | | 41.11 | | 57.05 | | 8.94E-06 | | 0.25 | |  |
| 2019_8_D | | 1 | 18.32 | | 0 | | 69.25 | | 4.28E-01 | | 0.29 | |  |
| 2019_10_D | | 3 | 61.03 | | 40.04 | | 65.12 | | 1.27E-04 | | 0.27 | |  |
| 2019_11_D | | 5 | 67.03 | | 46.09 | | 82.76 | | 2.30E-04 | | 0.18 | |  |
| 2019_D | | 1 | 46.01 | | 0 | | 69.25 | | 4.27E-01 | | 0.21 | |  |
| 2020_5_D | | 1 | 11.36 | | 0 | | 20.06 | | 7.43E-07 | | 0.18 | |  |
| 2020_5_D | | 3 | 51.14 | | 44.03 | | 51.14 | | 9.46E-11 | | 0.35 | |  |
| 2020_5_D | | 5 | 47.15 | | 43.1 | | 53.02 | | 9.98E-05 | | 0.14 | |  |
| 2020_6_D | | 1 | 30.25 | | 1.01 | | 32.03 | | 1.21E-06 | | 0.21 | |  |
| 2020_6_D | | 3 | 43.02 | | 36.08 | | 57.05 | | 1.05E-05 | | 0.18 | |  |
| 2020_6_D | | 5 | 43.1 | | 40.15 | | 70.04 | | 3.73E-04 | | 0.14 | |  |
| 2020_8_D | | 3 | 63.69 | | 52.2 | | 65.12 | | 1.65E-04 | | 0.21 | |  |
| 2020_D | | 3 | 51.14 | | 39.23 | | 57.05 | | 9.78E-07 | | 0.34 | |  |
| 2021_5_D | | 3 | 51.14 | | 32.13 | | 53.14 | | 3.50E-06 | | 0.2 | |  |
| 2021_5_D | | 5 | 47.15 | | 46.09 | | 47.15 | | 1.51E-09 | | 0.36 | |  |
| 2021_7_D | | 5 | 3.26 | | 0 | | 45.15 | | 3.17E-04 | | 0.18 | |  |
| 2021_7_D | | 7 | 5.16 | | 0 | | 12 | | 3.38E-04 | | 0.16 | |  |
| 2021_D | | 5 | 47.15 | | 24.12 | | 53.02 | | 1.85E-04 | | 0.23 | |  |
| overall_BLUEs_D | | 3 | 53.14 | | 43.02 | | 64.2 | | 1.64E-04 | | 0.3 | |  |

^a^ Blackspot (B), cercospora (C), and defoliation (D) are denoted after the year_month (i.e. 2019_6_B denotes blackspot measured in June of 2019).

^b^ Percent variance explained (PVE) is estimated in QTLpoly as the QTL heritability ($h_{q}^{2}$) as the ratio of between the variance attributed to the QTL and the total variance.

**Supplementary Table 2**. QTL for all black spot, cercospora, and defoliation for the garden rose tetraploid population Stormy Weather x Brite Eyes phenotyped over 3 years in Somerville, TX. BLUEs were calculated monthly, yearly, and overall.

| Trait ^a^ | Linkage group | Position (cM) | Lower interval (cM) | Upper interval (cM) | p-value | h^2 b^ |
| --- | --- | --- | --- | --- | --- | --- |
| 2019_6_B | 5 | 61.23 | 45.07 | 72.36 | <2.22e-16 | 0.66 |
| 2019_7_B | 5 | 61.23 | 45.07 | 72.36 | <2.22e-16 | 0.66 |
| 2019_8_B | 4 | 56.16 | 52.03 | 70.47 | 2.11E-04 | 0.15 |
| 2019_8_B | 5 | 61.23 | 35.08 | 67.08 | 1.11E-04 | 0.19 |
| 2019_9_B | 1 | 16.01 | 9.11 | 36 | 2.26E-04 | 0.2 |
| 2019_9_B | 5 | 58.33 | 54.02 | 70.06 | 6.39E-06 | 0.2 |
| 2019_10_B | 5 | 58.33 | 55.41 | 62.09 | <2.22e-16 | 0.49 |
| 2019_11_B | 2 | 26.02 | 24.03 | 40.01 | 3.56E-05 | 0.09 |
| 2019_11_B | 5 | 61.23 | 45.07 | 73.2 | <2.22e-16 | 0.62 |
| 2019_B | 5 | 61.23 | 38.06 | 72.36 | <2.22e-16 | 0.73 |
| 2020_5_B | 5 | 61.23 | 58.33 | 62.09 | <2.22e-16 | 0.49 |
| 2020_6_B | 5 | 31.01 | 23.2 | 45.07 | 7.87E-05 | 0.26 |
| 2020_B | 4 | 57.11 | 56.16 | 64.07 | 1.52E-04 | 0.12 |
| 2021_5_B | 5 | 61.23 | 58.33 | 67.08 | <2.22e-16 | 0.46 |
| 2021_6_B | 3 | 32.11 | 24.13 | 42.02 | 1.81E-04 | 0.22 |
| 2021_7_B | 2 | 34.02 | 29.02 | 44.1 | 1.90E-04 | 0.2 |
| 2021_8_B | 2 | 11.04 | 1.35 | 23.04 | 2.71E-04 | 0.21 |
| 2021_B | 2 | 33.04 | 28.12 | 39.05 | 1.58E-04 | 0.18 |
| overall_BLUEs_B | 1 | 21.14 | 9.11 | 40.04 | 1.77E-04 | 0.15 |
| overall_BLUEs_B | 5 | 61.23 | 55.41 | 68.01 | <2.22e-16 | 0.5 |
| 2019_6_C | 1 | 24.06 | 12 | 25.23 | 1.18E-06 | 0.1 |
| 2019_6_C | 5 | 58.33 | 48.04 | 71.02 | <2.22e-16 | 0.6 |
| 2019_7_C | 5 | 59 | 50.05 | 72.36 | <2.22e-16 | 0.64 |
| 2019_8_C | 1 | 24.06 | 23.31 | 25.23 | 2.49E-06 | 0.1 |
| 2019_8_C | 2 | 22 | 14.08 | 58.12 | 1.22E-04 | 0.08 |
| 2019_8_C | 5 | 61.23 | 53.01 | 64.02 | <2.22e-16 | 0.49 |
| 2019_9_C | 1 | 24.06 | 23.31 | 24.06 | 5.36E-07 | 0.18 |
| 2019_9_C | 5 | 61.23 | 50.05 | 68.01 | 4.61E-06 | 0.2 |
| 2019_10_C | 4 | 38.01 | 25.01 | 47.22 | 1.60E-04 | 0.15 |
| 2019_10_C | 5 | 26.15 | 19.29 | 74.07 | 8.81E-05 | 0.14 |
| 2019_11_C | 5 | 61.23 | 55.41 | 61.23 | 2.68E-10 | 0.41 |
| 2019_C | 1 | 24.06 | 21.14 | 25.23 | 4.81E-06 | 0.08 |
| 2019_C | 5 | 59 | 49.1 | 70.06 | <2.22e-16 | 0.58 |
| 2020_5_C | 1 | 24.06 | 12 | 25.23 | 4.22E-06 | 0.15 |
| 2020_5_C | 5 | 61.23 | 49.1 | 70.06 | <2.22e-16 | 0.52 |
| 2020_6_C | 1 | 24.06 | 20.35 | 25.23 | 2.60E-05 | 0.1 |
| 2020_6_C | 5 | 61.23 | 49.1 | 70.06 | <2.22e-16 | 0.53 |
| 2020_7_C | 5 | 67.08 | 54.02 | 71.02 | 9.70E-07 | 0.23 |
| 2020_C | 1 | 24.06 | 23.31 | 24.06 | 1.74E-07 | 0.13 |
| 2020_C | 4 | 25.01 | 10.98 | 30.04 | 3.05E-04 | 0.07 |
| 2020_C | 5 | 61.23 | 49.1 | 69.27 | <2.22e-16 | 0.48 |
| 2021_5_C | 1 | 24.06 | 16.01 | 25.23 | 3.71E-05 | 0.12 |
| 2021_5_C | 5 | 59 | 55.41 | 64.02 | 2.39E-07 | 0.26 |
| 2021_6_C | 1 | 24.06 | 0 | 69.1 | 4.23E-01 | 0.17 |
| 2021_7_C | 1 | 24.06 | 16.01 | 26.05 | 1.07E-04 | 0.11 |
| 2021_7_C | 2 | 20.11 | 16.07 | 33.04 | 1.11E-04 | 0.12 |
| 2021_7_C | 5 | 62.09 | 54.02 | 72.36 | 1.21E-05 | 0.18 |
| 2021_C | 1 | 24.06 | 19.06 | 27.04 | 8.64E-05 | 0.12 |
| 2021_C | 5 | 64.02 | 55.41 | 72.36 | 2.74E-05 | 0.18 |
| overall_BLUEs_C | 1 | 24.06 | 23.31 | 24.06 | 2.26E-08 | 0.13 |
| overall_BLUEs_C | 4 | 25.01 | 17.11 | 40.01 | 2.00E-04 | 0.07 |
| overall_BLUEs_C | 5 | 61.23 | 50.05 | 69.27 | <2.22e-16 | 0.47 |
| 2019_6_D | 5 | 61.23 | 45.07 | 72.36 | <2.22e-16 | 0.65 |
| 2019_7_D | 5 | 61.23 | 45.07 | 71.02 | <2.22e-16 | 0.59 |
| 2019_8_D | 5 | 56.23 | 47.18 | 57.04 | 1.42E-10 | 0.4 |
| 2019_9_D | 3 | 21.07 | 13.04 | 55.07 | 1.20E-04 | 0.17 |
| 2019_10_D | 3 | 24.13 | 20.36 | 32.11 | 2.11E-10 | 0.37 |
| 2019_11_D | 3 | 22.07 | 21.07 | 29.15 | 3.95E-10 | 0.29 |
| 2019_11_D | 5 | 55.41 | 55.41 | 61.23 | 4.95E-08 | 0.25 |
| 2019_D | 3 | 21.07 | 19.28 | 29.15 | 4.31E-06 | 0.14 |
| 2019_D | 5 | 56.23 | 55.41 | 63.11 | <2.22e-16 | 0.42 |
| 2020_5_D | 5 | 61.23 | 46.18 | 73.2 | <2.22e-16 | 0.79 |
| 2020_6_D | 5 | 67.08 | 65.1 | 67.08 | <2.22e-16 | 0.43 |
| 2020_7_D | 2 | 37.07 | 33.04 | 42.02 | 1.68E-05 | 0.21 |
| 2020_7_D | 7 | 46.02 | 42.21 | 52.34 | 2.12E-04 | 0.14 |
| 2020_8_D | 3 | 21.07 | 18.18 | 25.01 | 2.62E-06 | 0.24 |
| 2020_D | 5 | 61.23 | 55.41 | 69.27 | <2.22e-16 | 0.56 |
| 2021_5_D | 5 | 61.23 | 47.18 | 72.36 | <2.22e-16 | 0.69 |
| 2021_6_D | 2 | 54.17 | 48.95 | 64.26 | 2.72E-04 | 0.16 |
| 2021_6_D | 3 | 24.13 | 18.18 | 29.15 | 4.62E-05 | 0.18 |
| 2021_7_D | 5 | 55.41 | 55.41 | 61.23 | 6.33E-05 | 0.24 |
| 2021_D | 5 | 61.23 | 60.05 | 61.23 | <2.22e-16 | 0.43 |
| overall_BLUEs_D | 3 | 21.07 | 11.06 | 25.01 | 6.88E-06 | 0.11 |
| overall_BLUEs_D | 5 | 61.23 | 48.04 | 67.08 | <2.22e-16 | 0.53 |

^a^ Blackspot (B), cercospora (C), and defoliation (D) are denoted after the year_month (i.e. 2019_6_B denotes blackspot measured in June of 2019).

^b^ Percent variance explained (PVE) is estimated in QTLpoly as the QTL heritability ($h_{q}^{2}$) as the ratio of between the variance attributed to the QTL and the total variance.
